# Tuning the Electronic Properties of Tetravalent Cerium Complexes via Ligand Derivatization

**DOI:** 10.1021/acs.inorgchem.4c05371

**Published:** 2025-03-24

**Authors:** Georgilett Pérez Bedwell, Nithin Suryadevara, Zhibo Qi, Robert W. Gable, Peter Bencok, Michael L. Baker, Colette Boskovic

**Affiliations:** † School of Chemistry, University of Melbourne, Parkville, Victoria 3010, Australia; ‡ Department of Chemistry, The University of Manchester, Manchester M13 9PL, U.K.; § The University of Manchester at Harwell, Diamond Light Source, Harwell Campus, Didcot OX11 0DE, U.K.; ∥ Diamond Light Source, Harwell Science and Innovation Campus, Chilton, Didcot OX11 0DE, U.K.

## Abstract

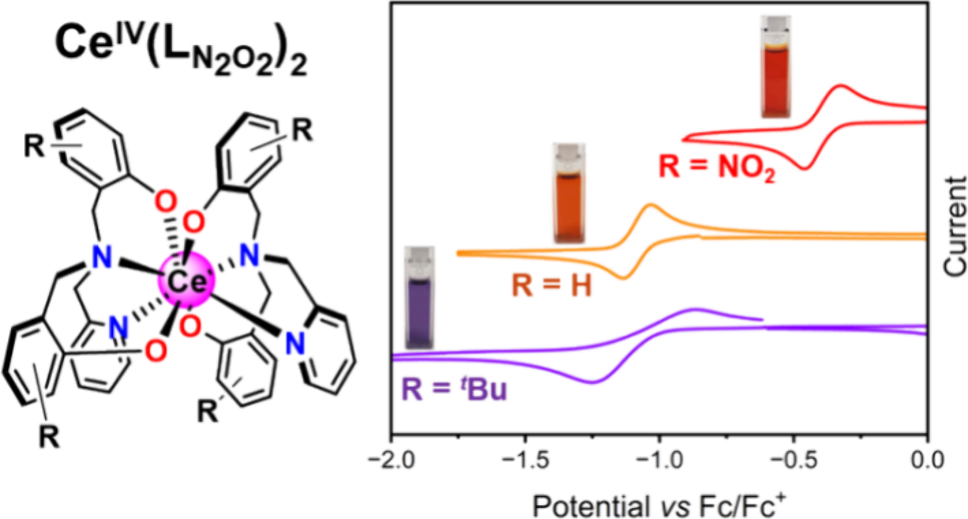

Molecular cerium complexes are of interest due to their remarkable redox and photophysical properties. We have investigated the ligand tunability of the electronic structure and properties of cerium(IV) complexes with functionalized tetradentate N_2_O_2_-donor ligands: [Ce^IV^(L_tBu_)_2_] **(1)**, [Ce^IV^(L_H_)_2_] **(2)** and [Ce^IV^(L_NO2_)_2_] (**3**), where H_2_L_tBu_ = bis(2-hydroxy-3,5-di-*tert*-butylbenzyl)(2-pyridylmethyl)amine, H_2_L_H_ = bis(2-hydroxybenzyl)(2-pyridylmethyl)amine and H_2_L_NO2_ = bis(2-hydroxy-5-nitrobenzyl)(2-pyridylmethyl)amine. These compounds all exhibit a quasi-reversible one-electron reduction to cerium(III), with the redox potential correlating with the electron donor–acceptor characteristics of the ligand substituents. This correlation is rationalized by energy stabilization of the HOMO, as determined by density functional theory calculations, and is consistent with arene π → Ce 4f* ligand-to-metal charge transfer bands. The L_3_-edge XANES exhibits minimal variation in Ce 4*f* occupation for the three compounds, which suggests that the *4f* covalent character and composition of the ground-state character do not vary significantly across the series. However, M_4,5_-edge XAS shows charge transfer satellites that subtly differ in shape and energy, indicating small distinctions in ligand-to-metal charge transfer for the compounds, consistent with small differences in temperature-independent magnetism. The ability to modulate the redox and optical properties of cerium complexes through ligand derivatization highlights the potential for customizable molecular cerium catalysts and photocatalysts.

## Abstract 2

The electronic structure of cerium(IV) ions has been effectively tuned through ligand derivatization in homoleptic bis-tetradentate cerium(IV) complexes, enabling the modulation of redox and optical properties.

## Introduction

The relative accessibility of the cerium(IV/III) redox couple is unique among the 4*f* elements and is critical for real-world applications of cerium-containing ionic solids. For example, catalytic converters in petrol cars employ cerium oxide for oxygen storage and release to support the oxidation of CO to CO_2_.^1,2^ Ceric ammonium nitrate is widely used as an oxidant in synthetic organic chemistry and quantitative analysis.^3,4^ The desire to harness this redox capability at the molecular level has afforded recent interest in the synthesis and investigation of redox-active cerium complexes.^5−10^

Schelter, La Pierre, and others have investigated in detail the redox properties of cerium complexes with a large range of ligands, providing important insights into the dependence of the cerium(IV/III) redox potential on the ligand environment.^10−15^ These studies are relevant for *f*-element speciation and separations and the isolation of high oxidation state *f*-element complexes.^16−24^ This body of work has included the incorporation of redox-active organic ligands to explore further possibilities with redox cycling, organic transformations, and separations.^25−29^ The photophysical properties of cerium ions are also important, with cerium(III) compounds exhibiting electric dipole-allowed interconfigurational 4*f* → 5*d* transitions, which give rise to broad absorption and emission bands, short lifetimes, and high emission intensities.^30,31^ Thus, cerium(III) complexes have been reported to act as stoichiometric and catalytic photoreductants, including for hydrogen atom abstraction reactions.^32,33^ In contrast, cerium(IV) complexes typically exhibit intense and varied colors due to parity-allowed ligand-to-metal charge transfer (LMCT) transitions.

The electronic structure of nominally cerium(IV) compounds is complicated, involving multiconfigurational Ce(^IV^, *f*^0^)/Ce(^III^, *f*^1^) ground-state electronic configurations and metal–ligand covalency.^6,10^ Experimental evidence of covalency (i.e., the mixing of Ce 4*f* orbitals character into ligand valence orbitals) can be obtained from ligand K-edge X-ray absorption spectroscopy.^34,35^ Similarly, M_4,5_-edge and L_3_-edge X-ray absorption spectroscopies are applied to provide quantitative insight into the effective ground-state 4*f* electron occupation and the extent of metal–ligand covalency, supported by charge transfer multiplet theory and, more recently, ab initio wave function based calculations.^17,36−42^ Temperature-independent paramagnetism arises from the presence of low energy, open-shell triplet, excited states that permit the mixing of significant triplet character into the ground state singlet.^38−42^ Electronic absorption spectra are also informative and can be accurately predicted using time-dependent density-functional theory (TD-DFT) calculations.^13,39,43^

We have recently applied to 4*f* metal-ions our longstanding interest in ligand variation for tuning the redox potentials and electronic properties of 3*d* metals, initially exploring tetradentate N-donor ligands derived from tripicolyamine in europium(II) complexes.^44^ A shift to related O-donor-containing ligands suitable for cerium has prompted an investigation of a family of homoleptic complexes of cerium(IV) with tetradentate N_2_O_2_-donor ligands derived from bis(2-hydroxybenzyl)(2-pyridylmethyl)amine. These ligands have previously been employed in europium(II) and various trivalent lanthanoid complexes.^45−48^ Herein, we report the synthesis and investigation of three homoleptic cerium(IV) complexes with N_2_O_2_-donor ligands: [Ce^IV^(L_tBu_)_2_] (**1**), [Ce^IV^(L_H_)_2_] (**2**), and [Ce^IV^(L_NO2_)_2_] (**3**), where H_2_L_tBu_ = bis(2-hydroxy-3,5-di-*tert*-butylbenzyl)(2-pyridylmethyl)amine, H_2_L_H_ = bis(2-hydroxybenzyl)(2-pyridylmethyl)amine and H_2_L_NO2_ = bis(2-hydroxy-5-nitrobenzyl)(2-pyridylmethyl)amine (Figure S1).

## Results and Discussion

### Synthesis

**Figure 1:**
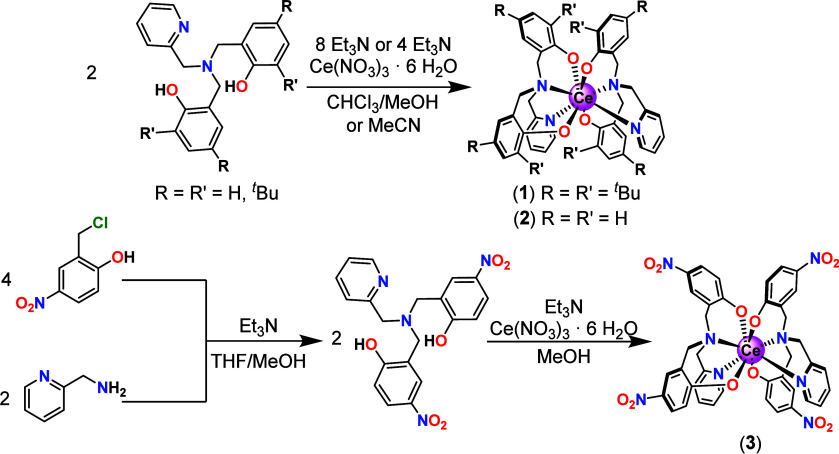
Synthetic pathways for compounds **1**, **2** (top), and **3** (bottom).

Neutral homoleptic complexes [Ce^IV^(L_tBu_)_2_] (**1**) and [Ce^IV^(L_H_)_2_] (**2**) were prepared (see Experimental Section) by reacting one equivalent of Ce(NO_3_)_3_ with two equivalents of ligand doubly deprotonated with Et_3_N at room temperature under ambient conditions (Figure 1). Complete deprotonation of the ligands was required to form the complexes. Complex [Ce^IV^(L_NO2_)_2_] (**3**) was synthesized by a modified two-step complexation reaction reported in the literature for copper and iron complexes.^49^ The ligand was synthesized in situ by reflux of two equivalents of 2-aminomethylpyridine in THF, four equivalents of 2-chloromethyl-4-nitrophenol in MeOH, and four equivalents of Et_3_N. The resulting solution was heated to reflux, filtered, and evaporated under reduced pressure to obtain the product as a gold-colored suspension. The ligand suspension was redissolved in MeOH and deprotonated with four equiv of Et_3_N. The addition of one equivalent of Ce(NO_3_)_3_ in MeOH afforded **3**. Crystals of **1** and **2** suitable for structure determination were obtained directly from the reaction mixture, while crystals of **3**·0.75CH_2_Cl_2_·H_2_O were obtained following recrystallization from CH_2_Cl_2_/MeOH.

Bulk crystalline samples were obtained in relatively good yields for all compounds, and purity was confirmed by elemental analysis (EA) and powder X-ray diffraction (Figure S2). Thermogravimetric (Figure S3) and elemental analysis of the bulk samples confirmed no solvation for **1** and **2** and some hygroscopicity for **3**, which analyses as **3**·0.8CH_2_Cl_2_·1.5H_2_O. The compounds are brightly colored: purple (**1**), orange (**2**) and red (**3**), consistent with Ce(IV). It is common for cerium reactions conducted under aerobic conditions to involve aerial oxidation of Ce(III) to Ce(IV).^39,50−54^

### Structure Description

**Figure 2:**
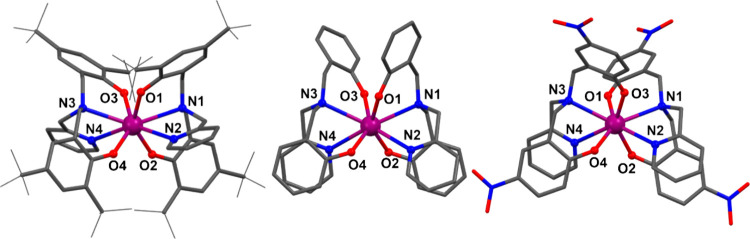
Molecular structures of **1** (left), **2** (middle), and **3** (right). Solvent molecules and hydrogen atoms were omitted for clarity. C: gray, N: blue, O: red, and Ce: magenta.

The solid-state structures for compounds **1**, **2,** and **3**·0.75CH_2_Cl_2_·H_2_O were determined by single crystal X-ray diffraction at 100 K (Figure 2 and Table S1). Compound **1** crystallizes as dark-purple rectangular blocks in monoclinic space group *I*_2_/*a*. The asymmetric unit consists of half of a molecule of the cerium compound. Compounds **2** and **3**·0.75CH_2_Cl_2_·H_2_O crystallize as dark-orange and red rectangular blocks, respectively, in the monoclinic space group *P*2_1_/*c*. For **2,** the asymmetric unit consists of two independent molecules of the Ce complex. The asymmetric unit for **3**·0.75CH_2_Cl_2_·H_2_O is composed of one molecule of the cerium complex and the solvent molecules.

In all three compounds, the Ce atom binds to two tetradentate L^2–^ ligands and is 8-coordinate. Continuous shape measures (Table S2) using the program SHAPE 2.1 indicate that the coordination geometry (Figure S4) in all cases is best described as snub disphenoid J84 (JSD-8).^55^ The two independent molecules **2**^**a**^ and **2**^**b**^ in **2** differ in the orientation of one of the phenyl rings on each ligand (Figure S5). The Ce–O/N bond lengths are similar for all complexes, with no clear correlation with the Hammett σ-parameters for the aryl substituents (Table 1, Figure S6), and are characteristic of Ce(IV) compounds.^54,56^ Bond valence sum calculations for all complexes are consistent with Ce(IV) (Table S3).^57,58^ The nearest intermolecular Ce···Ce distance (Table 1) is shorter for **2** (9.7652 Å), than for **1** (10.8079 Å) and **3** (11.0160 Å).

**Table 1:** Selected Interatomic Distances (Å) for **1**, **2,** and **3**·0.75CH_2_Cl_2_·H_2_O

	1	2a,b	2a,b	3·0.75CH_2_Cl_2_·H_2_O
Ce–O1	2.247(3)	2.1996(19)	2.1775(18)	2.208(2)
Ce–O2	2.189(3)	2.1869(19)	2.1848(19)	2.210(2)
Ce–O3	2.247(3)	2.1991(19)	2.1763(19)	2.179(2)
Ce–O4	2.189(3)	2.1856(19)	2.2122(19)	2.201(3)
Ce–N1	2.668(4)	2.718(2)	2.696(2)	2.682(3)
Ce–N2	2.761(4)	2.598(2)	2.651(2)	2.591(3)
Ce–N3	2.668(4)	2.718(2)	2.727(2)	2.674(3)
Ce–N4	2.761(4)	2.615(2)	2.682(2)	2.591(3)
Ce···Ce^c^	10.8079(4)	9.7652(5)		11.0160(6)

a,b Two independent molecules in **2**.

c Nearest intermolecular distance.

### Infrared Spectroscopy

The infrared spectra for **1**, **2,** and **3**·0.8CH_2_Cl_2_·1.5H_2_O were collected in the solid state (4000–400 cm^–1^) (Figure S7). The three compounds show similar vibrations, with slight variations related to the substituents in the coordinated ligands. Characteristic aromatic C–C stretching is observed for all compounds at ν̅ ∼ 1600 (m) cm^–1^. Bands observed in the range 1235–1277 cm^–1^ are assigned to C–N stretches of the benzylic amines along with other vibrations. For compound **3**, the N–O stretch is present at ν̅ ∼ 1500 (m) and ν̅ ∼ 1338 (w).^59^ Similarly, the *tert-*butyl stretch is observed for compound **1** in the region ν̅ ∼ 2948–2823 cm^–1^.

### Magnetic Measurements

**Figure 3:**
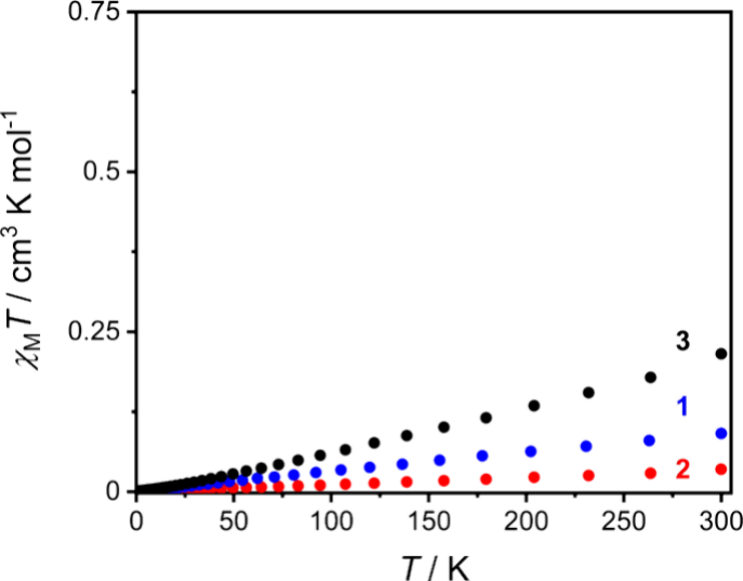
Plots of χ_M_*T* versus *T* for **1** (blue), **2** (red), and **3**·0.8 CH_2_Cl_2_·1.5H_2_O (black) with an applied field of 0.1 T.

Magnetic measurements were conducted on the three compounds to confirm the oxidation state and explore the magnetic behavior. Literature studies on Ce(IV) complexes have shown dominant Van Vleck temperature independent paramagnetism (TIP), which arises from the small energy gap between the open-shell singlet ground state and low-lying triplet excited states.^39,40^ Magnetic data were acquired for microcrystalline samples of **1, 2,** and **3**·0.8 CH_2_Cl_2_·1.5H_2_O loaded into gelatin capsules with a small amount of eicosane to prevent sample movement while using the most sensitive vibrating sample magnetometer (VSM) mode of the SQUID (see Experimental Section). Variable temperature molar magnetic susceptibility (χ_M_) data were measured with an applied magnetic field of 1000 Oe upon heating from 2 to 300 K. Diamagnetic corrections were made for the samples, eicosane, and sample holder, and the results of several measurements were averaged.^40,60^ All three compounds show a weak paramagnetic response (Figures 3 and S8), consistent with previous reports for Ce(IV) complexes,^38,40^ attributed to TIP and a small fraction of paramagnetic impurity. The χ_M_ versus *T* curves for **1**, **2**, and **3**·0.8CH_2_Cl_2_·1.5H_2_O were fit to a Curie–Weiss + constant model to determine values for the Curie constant (*C*_J_), the Curie–Weiss temperature (θ_CW_) and the level of TIP (Table S4). The values of *C*_J_ = (8.77 ± 2.16) × 10^–5^ emu K mol^–1^ (**1**), (7.09 ± 1.19) × 10^–5^ emu K mol^–1^ (**2**) and (2.18 ± 0.05) × 10^–3^ emu K mol^–1^ (**3**); (for comparison, *C*_5/2_ expected for a Ce(III) impurity is 0.807 emu K mol^–1^), suggest ∼0.01% of paramagnetic impurity for **1** and **2**, and less than 0.30% for **3**. The obtained values of χ_TIP_ = (3.12 ± 0.06) × 10^–4^ emu mol^–1^ (**1**), (1.48 ± 0.02) × 10^–4^ emu mol^–1^ (**2**), and (6.15 ± 0.04) × 10^–4^ emu mol^–1^ (**3**) are comparable to values previously reported for molecular tetravalent cerium complexes.^38−40^

### Electronic Spectroscopy

**Figure 4:**
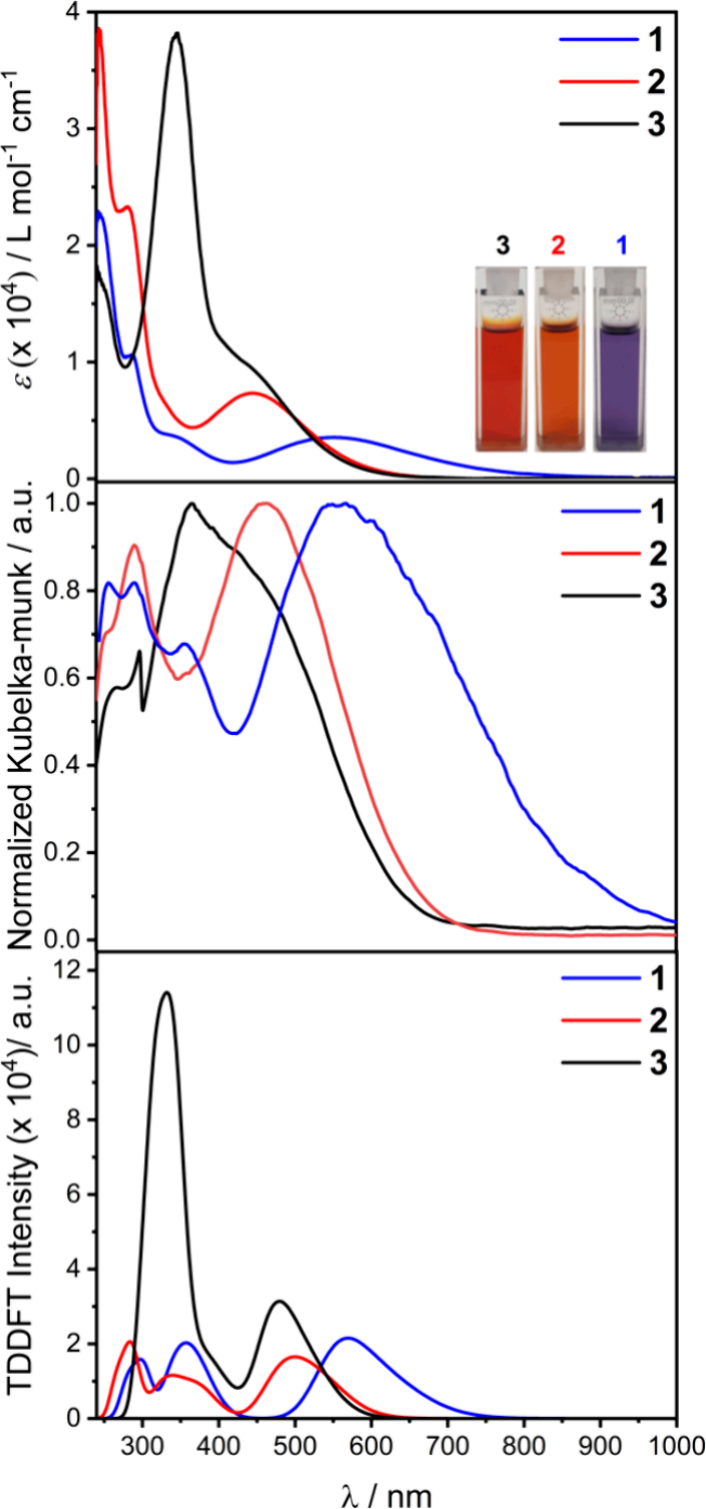
Electronic spectra of **1** (blue), **2** (red), and **3** (black) as absorption in CHCl_3_ with photographs of the solutions as an inset (top); as diffuse reflectance for a ∼5% diluted sample in KBr plotted as the Kubelka–Munk function (middle) and TDDFT-calculated spectra (bottom).

Electronic properties were examined in the solid state by diffuse reflectance measurements (Figure 4) in ∼5% in KBr. The collected spectra in the range of 200–1100 nm were processed as normalized Kubelka–Munk functions.^61^ Absorption spectra were measured in a CHCl_3_ solution (Figure 4), with spectra measured over a period of several hours remaining unaltered, confirming the stability of the compounds in solution (Figure S9). A consistent set of bands is observed for each complex between the solid and solution states (Table 2), with a blue shift of between 5 and 20 nm attributed to a solvent effect.^62^ The interaction between the solvent and the solute, viscosity, and dielectric constant of the solvents are responsible for the shifts, which are more evident in polar solvents.^62,63^ Spectra were assigned following simulation by time-dependent density functional theory (TDDFT) calculations, with excellent agreement obtained between the measured and calculated spectra for both peak positions and relative intensities (Figure 4).

**Table 2:** Electronic Spectral Data (λ /nm (ε/L·mol^–1^·cm^–1^) in CHCl_3_ and the Solid State for **1**, **2,** and **3**^a^

**1**			**2**			**3**			
solution	solid	calc.	solution	solid	calc.	solution	solid	calc.	assignment
552 (3.45 × 10^3^)	558	569.4 (0.0952)	445 (7.33 × 10^3^)	464	500.7 (0.0318)	∼437 (9.86 × 10^3^)	442	478.8 (0.1320)	**1**: aryloxide O lone pair → O 2*p -* Ce 4f* (LMCT)
									**2:** aryloxide O lone pair → O 2*p -* Ce 4f* (LMCT)
									**3**: NO_2_-aryloxide O lone pair → O 2*p -* Ce 4f* (LMCT)
						407 (1.18 × 10^4^)	416 (sh)^b^	379.6 (0.0897)	O/N 2*p* - Ce 4*f*/5*d* → O 2*p -* Ce 4f* (LMCT)
343 (3.80 × 10^3^)	356	356.8 (0.0698)	335 (6.09 × 10^3^)	358, 370	339 (0.0177), 372 (0.0216)	347 (3.81 × 10^4^)	366	332.1 (0.2409)	**1:** O 2*p* – Ce 4*f*/5*d* → O 2*p* - Ce 4f* (LMCT)
									**2**: arene π – O/N 2*p* → O 2*p* - Ce 4f* (LMCT) & O/N 2*p* - Ce 4*f* → O 2*p* - Ce 4f* (LMCT)
									**3**: arene – NO_2_/O π → NO_2_/O π* (LLCT)
286 (1.06 × 10^4^)	260, 292	296.9 (0.0454)	282 (2.33 × 10^4^)	290	283.6 (0.0430)				**1**: O/N 2*p* - Ce 4*f*/5*d* → O 2*p* – Ce 4f* (LMCT)
									**2**: O/N 2*p* - Ce 5*d* → O 2*p* - Ce 4f* (LMCT)

a The TDDFT transition energies are given for transitions with the most significant oscillator strengths (intensities are given in brackets).

b sh = shoulder.

**Figure 5:**
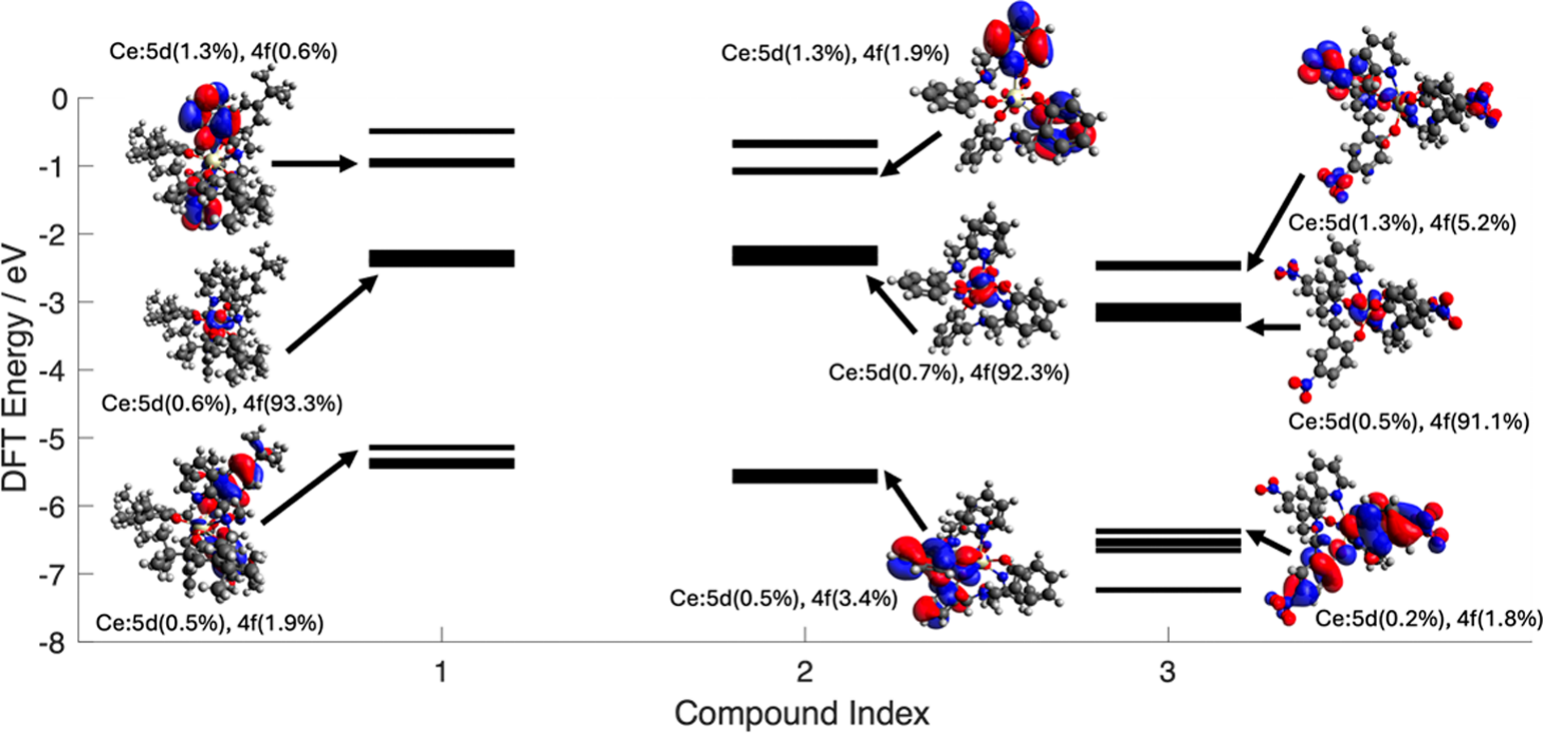
Valence molecular orbital diagrams for **1**, **2**, and **3** obtained from DFT calculations. The calculated HOMO–LUMO energy gap is 2.70 eV for **1**, 3.07 eV for **2,** and 3.12 eV for **3**.

All compounds exhibit multiple intense bands in the UV–visible region (Figure 4), affording the bright colors of the Ce(IV) compounds. Overall, bands in the UV region (λ < 330 nm) can be loosely assigned as O/N 2*p* – Ce 5*d* → Ce 4f* ligand to metal charge transfer (LMCT) transitions, although this band is not evident for **3**, as it likely lies at higher energy. Bands in the visible region are aryloxide lone pair → Ce 4f* ligand to metal charge transfer (LMCT) transitions from different bonding orbitals. Detailed assignments are given in Table 2 and corresponding molecular orbitals are shown in Figures S10–S12. The most intense LMCT bands shift to high energy in the order **1** < **2** < **3** and there is a clear correlation with the Hammett σ-parameters for the aryl substituents (Figure S13). This reflects the lowering in energy of HOMOs in the case of more electron-withdrawing substituents on the ligand (Figure 5).

### L_3_-Edge X-ray Absorption Fine Structure Spectroscopy and M_4,5_-Edge X-ray Absorption Spectroscopy

**Figure 6:**
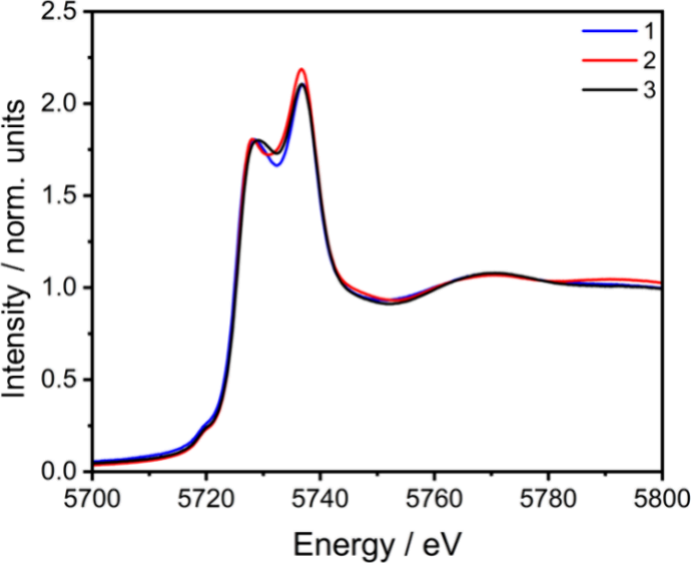
Ce L_3_-edge XANES spectra of **1**, **2,** and **3**·0.8 CH_2_Cl_2_·1.5H_2_O.

The L_3_-edge XANES of **1**, **2,** and **3**·0.8 CH_2_Cl_2_·1.5H_2_O are shown in Figure 6. The spectra are very similar, with low-intensity 2*p* → 4*f* pre-edge features centered at 5717.0 eV and two intense 2*p* → 5*d* bands of absorptions centered in energy at approximately 5726.0 and 5734.5 eV. The observed energy splitting of L_3_-edge XANES is typical for Ce(IV) compounds.^34,38−42,64−66^ The higher energy peak corresponds to the formal Ce(IV) *2p*^*6*^*4f*^*0*^*5d*^*0*^ → *2p*^*5*^*4f*^*0*^*5d*^*1*^ transitions, and the lower energy (∼5726.0 eV) peak is associated with absorption final state configurations that include a ligand to Ce *4f* charge transfer (2p^5^*4f*^*1*^L*5d*^*1*^), where *L* represents a ligand hole.^67^ The amount of Ce(III) (*4f*^*1*^*L*) character in the ground state is estimated by taking the ratio of the fitted peak intensities for the lower energy peak relative to the total edge intensity. Figures S14–S16 present the fitted peak analyses, and the effective 4*f* electron occupation (*n*_*4f*_) is given in Table 3. Alongside the experimentally determined 4*f* electron occupations, Table 3 shows calculated effective electron occupation obtained from analysis of ground state DFT. Both the L_3_-edge XANES and DFT analysis show insignificant variation in 4*f* electron population for compounds **1**, **2,** and **3**·0.8 CH_2_Cl_2_·1.5H_2_O with a greater 4*f* electron population from DFT.

**Table 3:** Ce Effective 4*f* (*n*_4*f*_), *5d* (*n*_5*d*_), and 6s (*n*_6*s*_) Electron Occupation Obtained from DFT Natural Atomic Orbital Occupancy and the Ce Effective 4*f* (*n*_4*f*_) Extracted from Ce L_3_-Edge XANES Peak Fitting Analysis

	**1**	**2**	**3**·0.8 CH_2_Cl_2_·1.5H_2_O
DFT *n*_6*s*_	0.1	0.13	0.13
DFT *n*_5*d*_	0.59	0.94	0.93
DFT *n*_4*f*_	0.83	0.84	0.84
XANES *n*_4*f*_	0.39 ± 0.04	0.41 ± 0.04	0.41 ± 0.03

**Figure 7:**
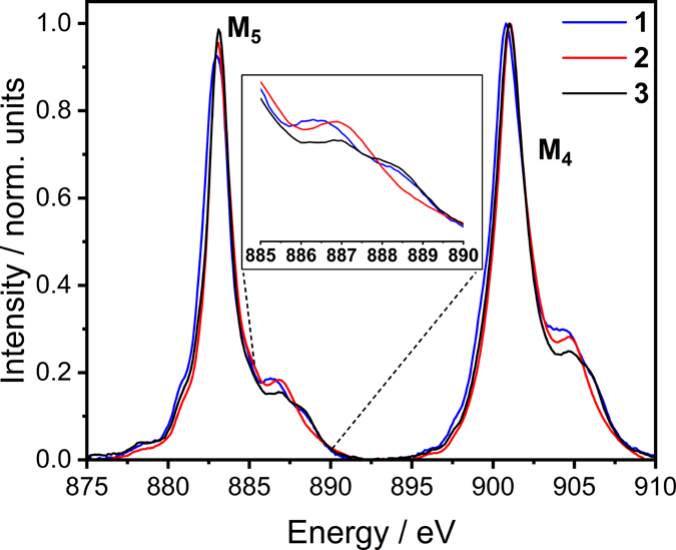
Overlay of X-ray absorption spectra of **1** (blue), **2** (red), and **3**·0.8 CH_2_Cl_2_·1.5H_2_O (black) at M_4,5_-edge normalized at the maximum of M_4_-edge intensity.

The background-subtracted M_4,5_-edge X-ray absorption spectra for compounds **1–3** are shown in Figure 7. The M_4,5_-edge includes two main absorption features at ∼ 883.1 eV and ∼ 901.0 eV that originate from 3*d*^9^ spin–orbit coupling, which splits excitations into the M_5_-edge (3*d*_5/2_ →4*f*) and M_4_-edge (3*d*_3/2_ →4*f*), respectively. At higher energy, satellite peaks are identified at ∼ 886.9 eV and ∼ 904.7 eV and assigned as ligand to metal charge transfer (LMCT) satellites.^41,42^ The charge transfer satellites exhibit a fine structure with variations in the energy and intensity, highlighted in the inset of Figure 7. Charge transfer multiplet theory calculations can be employed to correlate the energies and intensities of satellite peaks with the strengths of LMCT and *n*_4*f*_. The implementation of a charge transfer multiplet model (Anderson impurity model) for the simulation of M_4,5_-edge XAS of Ce(IV) requires two electron configurations in the XAS initial state separated by an energy Δ and in the XAS excited state by an energy Δ’. A configuration interaction parameter, *T* couples the configurations. In the initial state , where  represents the ligand valence shell following the donation of one electron and *Ĥ* is the molecular Hamiltonian. This method has succeeded when applied to simulate covalency contributions to many transitions metal L_2,3_-edge XAS compounds^68,69^ and the Ce M_4,5_-edge XAS spectra of high-symmetry compounds where charge transfer satellites do not exhibit the subtle fine structure observed in these examples.^34,41,42^ We found that it was not possible to apply such simulations to model the M_4,5_-edge spectra of **1**-**3** with the precision required to reproduce the relatively small differences observed experimentally. Accurate reproduction of the fine structure present within satellites **1** and **3** requires more than one charge transfer parameter (Δ) and *T* parameter in both ground and XAS final states, resulting in overparameterization that limits the insight that can be obtained from conducting such simulations.

### Electrochemistry

**Figure 8:**
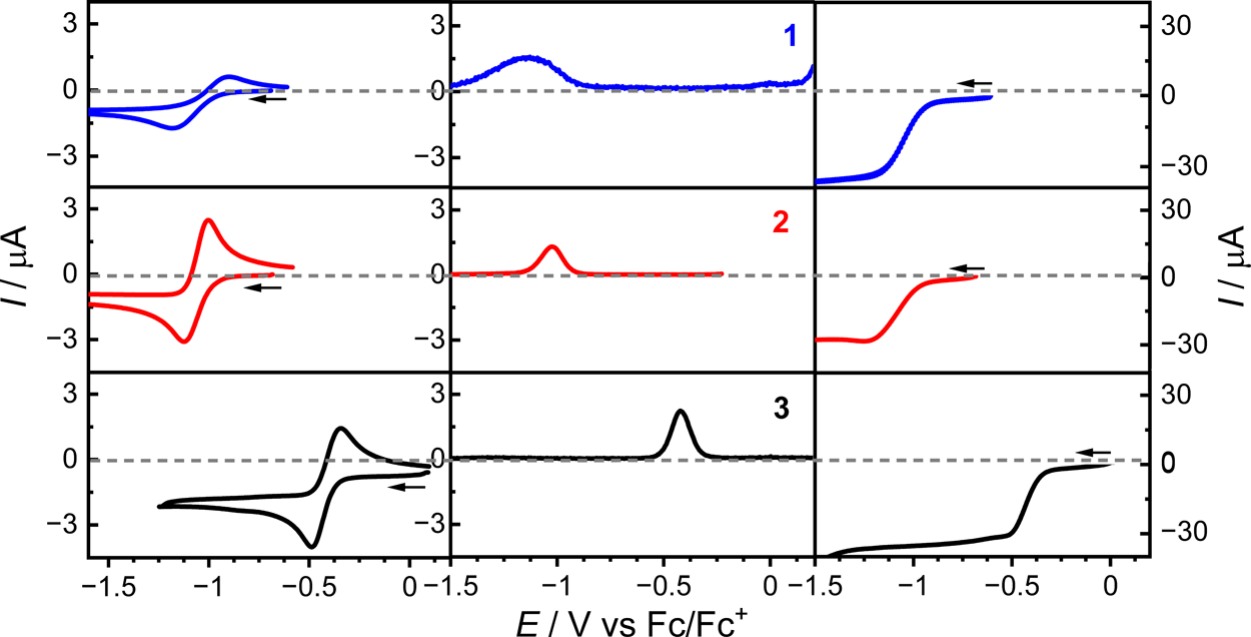
Cyclic (left), differential pulse (center), and rotating disk electrode (right) voltammograms for compounds **1–3** (1 mM in CH_2_Cl_2_ solution with 0.25 M Bu_4_NPF_6_ as the supporting electrolyte). CV conducted at a scan rate of 100 mV s^–1^, RDE at 500 rotations per minute and DPV at a scan rate of 10 mV s^–1^ with a pulse width of 500 ms.

To further investigate the effect of substituents on the ligands, the electrochemical properties for compounds **1–3** were measured with cyclic voltammetry (CV), differential pulse voltammetry (DPV), and rotary disk electrode (RDE) (Figures 8 and S17–S19). Measurements were conducted with 1 mM analyte solution in CH_2_Cl_2_ with 0.25 M Bu_4_NPF_6_ as supporting electrolyte, referenced to the ferrocene/ferrocenium (Fc/Fc^+^) couple. Electronic absorption spectroscopy over time confirms the solution stability of the complexes (Figure S8). The midpoint potentials (*E*_m_) were determined by taking the average of the peak anodic potential (*E*_pa_) and peak cathodic potential (*E*_pc_) from the CV (Table 4). The peak potential (*E*_*p*_) is reported in the case of irreversible processes. Where possible, the peak separation (Δ*E*_p_), between *E*_pa_ and *E*_pc_ is given. Rotating disk electrode voltammetry was used to confirm the nature of the processes as oxidations or reductions via the position of zero current. It was also used to characterize the half-wave potentials (*E*_1/2_) and limiting currents (*i*_L_) to determine the number of electrons involved in the redox process.^70−73^

**Table 4:** Cyclic and RDE Voltammetry Data for Compounds **1**, **2,** and **3** in CH_2_Cl_2_^a^

CV *E*_m_ or *E*_pa_/V (Δ*E*_p_/mV)					RDE *E*_1/2_/V (*i*_L_/μA)		
	I	II	III	IV	I	II	III
1	–1.052(210)	0.323(74)	0.685(75)	0.940(74)	–1.055(31)	0.327(33)	0.704(31)
2	–1.063(120)	0.507*	0.643*	0.893*	–1.067(28)		
3	–0.419(87)	1.212*			–0.423(31)		

a Potentials for all processes observed for **1**, **2**, and **3** (S17–S19) are reported vs ferrocene/ferrocenium couple. The CV and RDE measurements were performed at room temperature in a 1 mM CH_2_Cl_2_ solution with 0.25 M Bu_4_NPF_6_ as the supporting electrolyte. The CV was conducted at a scan rate of 100 mVs^–1^ and RDE at 500 rotations per minute. The Δ*E*_p_ is not included due to the irreversibility of the processes (*).

The voltammograms for **1**, **2,** and **3** display one reduction process (I) and one (**1**) or three (**2** and **3**) oxidation processes (II–IV), with the RDE position of zero current confirming these as reductions and oxidations. Process I is quasi-reversible, with Δ*E*_p_ from the CVs and the peak width in the DPVs decreasing in the order **1** > **2** > **3**. This process is assigned as one-electron Ce(IV) to Ce(III) reduction. The *E*_m_/*E*_1/2_ values vary from ∼−1.05 V for **1** and **2** to −0.42 V for **3**, consistent with most facile reduction in the case of the electron-withdrawing nitro substituents on L_NO2_^2–^ in **3** versus the electron-donating *tert*-butyl substituents of L_tBu_^2–^ in **1**.^16^ As expected, electron-withdrawing ligand substituents favor Ce(III), while electron-donating substituents favor Ce(IV) and there is a clear correlation with the Hammett σ-parameters for the aryl substituents (Figure S20). The measured shift in the redox potential of around 600 mV across the series is modest and consistent with the relatively subtle chemical variation across the three complexes. This shift is also aligned with the trends of substituent effects tuning redox potentials reported for Ce(IV) complexes of salen and tetrakis(pyridyl-nitroxide), with reported shifts of 260 and 480 mV in Ce(IV), respectively.^13,74^ These values fall around the midpoint of the wide range of 1.0 to −2.9 V reported for this process for literature Ce complexes in nonaqueous media, indicating that the ligands used in this work neither particularly stabilize nor destabilize Ce(IV) compared to previously employed ligands.^8,10,14,24,74^ The redox potentials of the studied complexes fall within the region reported for cerium complexes of anionic oxygen-based ligands—such as alkoxides, aryl oxides (including salen-type ligands), and β-diketonates, consistent with the similar coordination spheres.^14,74^

Compounds **1** and **2** exhibit three oxidation processes with an accessible potential window. The first two of these processes (II and III) are quasi-reversible for **1** and the RDE *i*_L_ values suggest these are one-electron oxidations by comparison with the Ce^IV^ to Ce^III^ reduction (process I). Process IV for **1** and all three processes for **2** are irreversible. Only a single irreversible oxidation (II) is evident for **3** within the potential window, which is shifted to more positive potentials compared to **1** and **2** and consistent with the shifts for reduction process I. These oxidation processes are assigned as ligand-based and assumed to occur in the phenolate groups.^75^ In principle, each of the four phenolate groups can be oxidized separately. The quasi-reversibility of the first two oxidation processes for **1** suggests a degree of stability for the one- and two-electron oxidized forms.

## Concluding Remarks

Herein we report a new family of homoleptic bis-tetradentate cerium(IV) complexes that feature N_2_O_2_-donor ligands. The ligands have been derivatized to investigate the electronic effects of electron-donating (*tert*-butyl) versus electron-withdrawing (nitro) substituents on the Ce(IV) electronic structure and resulting properties, redox, and spectroscopic properties. The electronic transitions have been characterized by electronic absorption and reflectance and X-ray spectroscopy and interpreted by TDDFT calculations. The L_3_-edge XANES and DFT natural bond orbital population analysis indicate insignificant variation in Ce 4*f* occupation over the series, which suggests that the multiconfigurational ground state and ligand-to-metal covalent character do not vary significantly over the series. However, the electronic spectra indicate that the most intense arene π → Ce 4f* LMCT band correlates with the Hammett σ-parameters for the ligand aryl substituents, occurring at lower energy for **1** versus those of **2** and **3**. This observation indicates that the HOMO–LUMO energy gap is smaller for **1** versus **2** and **3**, which is confirmed by DFT calculations. However, this energy difference does not strongly influence the 4*f* character, which is in contrast to the literature on Ce(IV) compounds, where low-energy LMCT bands have been shown to correlate with the effective 4*f* electron population.^39^

The variation in the electronic properties across the three compounds was further explored by electrochemistry. The ligand derivatization has an easily rationalized effect on the redox properties, with the potential of the reversible Ce(IV) to Ce(III) reduction process shifting by around 600 mV across the series. The electron-withdrawing nitro substituents in **3**, afford the least negative reduction potential, suggesting that this complex would be most easily reduced. This finding is consistent with DFT results where a significant energy stabilization of the HOMOs is identified in going from **2** to **3**, with the effective *4f* occupation remaining unaffected, according to DFT and L_3_-edge XANES.

This work demonstrates the power of a multi-technique approach to determine Ce(IV)/Ce(III) character. It is evident that X-ray spectroscopy is a powerful supplement to electronic spectroscopy with DFT calculations essential for data interpretation. In this work, the TIP measured by magnetometry does not correlate with the spectroscopic data and DFT calculations. This provides supporting evidence that TIP is sensitive to factors beyond effective *4f* occupation. Variation in ligand derivatization can cause significant changes in Ce *4f-5d* hybridization^40^ and the redistribution of underlying states beyond the predictability of systematic trends.

Previous studies have demonstrated that significant variation in coordination number and ligand donor characteristics strongly influences the cerium(IV) multiconfigurational character and HOMO–LUMO energy energies.^17,39^ In contrast, the more subtle ligand derivatization explored in this work supports earlier findings^13^ that the HOMO–LUMO energies can be fine-tuned without substantially altering the covalent character of the metal ion. Future work will explore heteroleptic complexes, particularly those featuring redox-active ligands, to further refine the use of ligand variation in tailoring the cerium electronic structure and its associated physical and chemical properties.

## Experimental Section

No uncommon hazards are noted.

### Materials

All reagents purchased were of reagent grade or higher and used without further purification. All of the reactions were performed aerobically. Ligands bis(2-hydroxybenzyl)(2-pyridylmethyl)amine (H_2_L_H_) and bis(2-hydroxy-3,5-di-*tert*-butylbenzyl)(2-pyridylmethyl)amine (H_2_L_tBu_), were synthesized according to the literature.^59,76^ All of the other chemicals were purchased from commercial suppliers. Ligand H_2_L_NO2_ was synthesized in situ in the synthesis of compound **3**.

### [Ce^IV^(L_tBu_)_2_] (**1**)

A solution of deprotonated ligand L_tBu_^2–^ was prepared by reacting an excess of Et_3_N (167 μL, 1.20 mmol) with H_2_L_tBu_ (164 mg, 0.301 mmol) in 1:1 CHCl_3_/MeOH (30.0 mL). The ligand solution was added to a solution of Ce(NO_3_)_3_·6H_2_O (65.1 mg, 0.150 mmol) in 1:1 CHCl_3_/MeOH (30.0 mL) The resulting solution turned faintly purple after 1 min of stirring, changing to a deep purple after 3 min. Dark purple block-shaped crystals of **1** suitable for structural characterization formed from a concentrated solution layered with MeOH after 1 week. A mixture of colorless ligand crystals and dark purple crystals was collected by filtration and washed with MeOH. A pure bulk sample was obtained by dissolving the product mixture in hot MeCN and filtering the solution while still hot to remove the ligand and leave **1** as a solid product. The bulk sample was isolated by filtration, washed with methanol, and air-dried. Yield: 103 mg, 0.0840 mmol, ∼56% based on Ce(NO_3_)_3_. Thermogravimetric and elemental analyses confirmed no solvation for the bulk sample of **1**. Anal. Calc for CeC_72_H_100_N_4_O_4_: C, 70.55; H, 8.22; N, 4.57. Found: C, 70.14; H, 8.25; N, 4.61. Selected IR data (ν̅/cm^–1^): 2950–2820 (m), 1601 (w), 1460 (m) 1435 (m), 1308 (m), 1238 (s), 824 (s), 740 (s), 523 (s), 422(s). ^**1**^**H NMR** (400 MHz, CDCl_3_): δ = 8.57 (d, 2H, py), 7.09 (d, 2H, aryl), 7.05 (dt, 2H, py), 6.95 (d, 2H, py), 6.94 (d, 2H, aryl), 6.81 (d, 2H, aryl), 6.63 (t, 2H, py), 6.29 (d, 2H, aryl), 5.58 (d, 2H, py-CH_2_), 4.51 (d, 2H, py-CH_2_), 3.71 (d, 2H, Ar–CH_2_), 3.55 (d, 2H, Ar–CH_2_), 3.50 (d, 2H, Ar–CH_2_), 3.44 (d, 2H, Ar–CH_2_), 1.40 (s, 18H, CH_3_ of *^t^*Bu), 1.38 (s, 18H, CH_3_ of *^t^*Bu), 1.21 (s, 18H, CH_3_ of *^t^*Bu), 1.14 (s, 18H, CH_3_ of *^t^*Bu) ppm.

### [Ce^IV^(L_H_)_2_] (**2**)

A solution of the deprotonated ligand L_H_^2–^ was prepared by reacting Et_3_N (41.7 μL, 0.600 mmol) with H_2_L_H_ (96.1 mg, 0.300 mmol) in MeCN (30.0 mL). The ligand solution was added dropwise to a solution of Ce(NO_3_)_3_·6H_2_O (65.1 mg, 0.150 mmol) in MeCN (30.0 mL) while stirring. The resulting solution turned pale orange after 1 min, then dark orange after 5 min. The solution was stirred for 1 h at room temperature, filtered, and evaporated to half of the initial volume under reduced pressure. Dark orange block-shaped crystals of **2** suitable for structural characterization formed after 1 week. The bulk sample was collected by filtration, washed with methanol, and air-dried. The thermogravimetric analysis confirmed no solvation for the bulk sample of **2**. Yield: 60.6 mg, 0.0780 mmol, ∼52% based on Ce(NO_3_)_3_. Anal. Calc. for CeC_40_H_36_N_4_O_4_: C, 61.83; H, 4.68; N 7.21. Found: C, 61.82; H, 4.84; N, 7.22. Selected IR data (ν̅/cm^–1^): ∼3050 (w), 2824 (w), 1590 (m), 1500 (m), 1450 (s), 1338 (w), 1265 (s), 886 (m), 748 (s), 586 (s), 468 (s). ^**1**^**H NMR** (400 MHz, CD_3_CN): δ = 9.41 (d, 1H, py), 9.32 (d, 1H, py), 7.58 (dt, 1H, aryl), 7.37 (dt, 1H, aryl), 7.21 (dd, 1H), 7.23 – 6.8 (m, 10H, py and aryl), 6.62 (d, 1H), 6.5 (q, 2H, py/aryl), 6.19 (d, 1H, py/aryl), 6.05 (dd, 4H, py/aryl), 5.45 (d, 1H, py-CH_2_), 5.10 (d, 1H, py-CH_2_), 4.25 (d, 1H, Ar–CH_2_), 3.80 (d, 2H, Ar–CH_2_), 3.62 (d, 2H, Ar–CH_2_) ppm.

### [Ce^IV^(L_NO2_)_2_] (**3**)

A solution of 2-aminomethylpyridine (96.9 μL, 1.00 mmol) in THF (12.0 mL) was added dropwise to 2-chloromethyl-4-nitrophenol (356 mg, 1.90 mmol) in MeOH (10.0 mL). Four eq of Et_3_N (279 μL, 2.00 mmol) were added to the resulting solution and it was then heated to reflux at 75 °C for 2 h. Charcoal was added, the solution was filtered, and the filtrate was evaporated under reduced pressure until a golden suspension was formed. The resulting suspension was dissolved in MeOH (8.00 mL). Four eq of Et_3_N were added (279 μL, 2.00 mmol) to deprotonate the ligand followed by a dropwise addition to a solution of Ce(NO_3_)_3_·6H_2_O (217 mg, 0.500 mmol) in MeOH (4.00 mL). The resulting solution was then stirred for 1 h. The crude product was filtered, redissolved in CH_2_Cl_2_ (4.00 mL), and layered with MeOH (4.00 mL) in a closed vial. Crystals for crystallographic data collection grew after 10 days by solvent diffusion and were kept in contact with the mother solution; they were identified crystallographically as **3**·0.75CH_2_Cl_2_·H_2_O. The bulk sample was isolated by filtration, washed with methanol, and air-dried. Yield: 296 mg, 0.280 mmol, ∼56% based on Ce(NO_3_)_3_. TGA and elemental analysis suggest the sample is hygroscopic, analyzing for **3***·*0.8CH_2_Cl_2_·1.5H_2_O Anal. Calc. for CeC_40_H_32_O_12_N_8_·0.8CH_2_Cl_2_·1.5H_2_O: C, 46.59; H, 3.51; N, 10.65. Found: C, 46.09; H, 3.01; N, 10.39. Selected IR data (ν̅/cm^–1^): ∼1600 (m), 1500 (m), 1387 (w), 1338 (m), 1278 (s), 1089 (m), 934 (m), 646 (m), 543(w), 456 (m), 727 (m), 464 (m). ^**1**^**H NMR** (400 MHz, CDCl_3_): δ = 9.32 (d, 4H, py), 7.67 (dt, 4H, py), 7.23 (m, 8H, aryl), 6.16 (d, 4H, aryl), 3.77 (m, 8H, aryl-CH_2_), 3.51 (m, 4H, py-CH_2_) ppm.

### Single Crystal and Powder X-ray Diffraction

The crystallographic data and PXRD for the three compounds were collected using a Rigaku XtaLAB Synergy, Dualflex, and HyPix X-ray diffractometer at 100 K (Cu Kα radiation, λ = 1.5418 Å). The data were reduced using CrysAlisPro software employing a numerical absorption correction based on Gaussian integration over a multifaceted crystal. Using OLEX2,^77^ the structures were solved with ShelXT,^78^ using intrinsic phasing, and refined with ShelXL^79^ using a least-squares minimization method based on *F*^2^. Non-hydrogen atoms were refined using anisotropic displacement factors, while hydrogen atoms were placed at geometrical estimates and refined using the riding model with an isotropic displacement parameter of 1.5Ueq of the parent atom, for all methyl carbon atoms, and 1.2Ueq of the parent atom, for all other atoms. For **3** the electron density map showed peaks due to diffuse solvent which could not be adequately modeled so the contribution of the diffuse solvent was modeled using the OLEX2 solvent mask routine, the electron density was consistent with the presence of 0.75 molecules of CH_2_Cl_2_, and one molecule of water per formula unit. Powder samples were prepared by grinding a few crystals of the bulk samples. Data were collected at 2θ = 60^ο^ with an exposure time of 70 s per frame.

### Infrared Spectroscopy

The infrared spectra (IR) for **1**, **2,** and **3**·0.8CH_2_Cl_2_·1.5H_2_O were collected in the solid state as transmittance on a Bruker Alpha spectrometer. The parameters were set to 50 scans, resolution = 4, and a window range of 4000–400 cm^–1^.

### Elemental Analyses

(CHN) were performed at Macquarie Analytical and Fabrication Facility, Macquarie University, Sydney, Australia.

### Thermogravimetric Analyses

(TGA) were performed under a N_2_ atmosphere using a ramp rate of 5 °C per minute reaching a maximum temperature of 400 °C.

### Electronic Spectroscopy

Agilent Technology Cary 60 UV–visible spectrometer was used for ultraviolet–visible light spectroscopy. Samples were dissolved in CHCl_3_ and measured in a 1.0 cm quartz cuvette at wavelengths ranging from 200 to 1000 nm. The stability of each sample in solution was measured over 4 h by collecting the spectra every 15 min. Diffuse reflectance. A small amount of sample was ground and diluted in KBR in a ∼5% ratio. The fine powder was placed into the quartz holder and the spectra were collected from 200 to 1100 nm with a bandwidth of 1 nm.

### Magnetic Measurements

Magnetic measurements were performed on polycrystalline samples using a Quantum Design MPMS-XL SQUID magnetometer operating between 1.8 and 400 K. The sample was weighed (15–20 mg) in a gelatin capsule, and a small quantity of melted eicosane was added to prevent movement during measurement using the vibrating sample magnetometer (VSM) mode, which is most sensitive for low-moment samples. The gelatin capsule was mounted on a plastic straw. Samples were centered at low temperature (2 K) for maximum sensitivity and magnetic susceptibility data were measured upon heating from 2 to 300 K with an applied field of 0.1 T. Diamagnetic corrections were applied using Pascal’s constants for the compounds and measured blanks for the eicosane and sample holder and the results of several measurements were averaged. The χ_Μ_ vs *T* plots were fit to the Curie–Weiss law + TIP (χ = (*C*_J_/*T −*θ_CW_) + χ_0_), for which the data collected from 38 to 57 K were omitted due to interference from a paramagnetic O_2_ impurity.

### Ce L_3_-Edge XANES Spectroscopy

XANES measurements were performed at B18^80^ at Diamond Light Source, U.K., in transmission mode at room temperature. Samples were prepared as pellets homogeneously diluted in cellulose to give an absorption of 0.5 at the Ce L_3_-edge. The excitation energy was selected by using a Si(111) monochromator. Repeated measurements on positions on the sample previously unexposed to X-rays were conducted to confirm that spectra are free of X-ray-induced changes in the oxidation state. Spectra were normalized to give a post-edge absorption of unity. The monochromator energy was calibrated to the first inflection of the L_3_-edge of a CeO_2_ reference sample by setting the maximum in the first derivative of the spectrum to 5723.0 eV.^81^ Peak fitting was conducted to determine effective *4f* electron occupation (*n*_*4f*_) with Pseudo-Voigt functions to reproduce the L_3_-edge fine structure. The postedge contribution at the higher energy side of the edge was fit with a step function and a negative Pseudo-Voigt function to reproduce oscillation in intensity above the edge.

### Ce M_4,5_-Edge XAS

Measurements were performed at the I10 electromagnet end station at Diamond Light Source, U.K. Samples were prepared by pasting an even layer of a power sample to double-sided carbon tape. Measurements were obtained by continuously scanning the monochromator energy over the Ce M_4,5_-edge with electron yield detection obtained via a drain current measurement. Measurements were conducted with circular polarized X-rays in no applied magnetic field. Repeated measurements on positions on the sample previously unexposed to X-rays were conducted to confirm that spectra are free of X-ray-induced changes in the oxidation state. Measurements were performed with the cryostat set to 20 K within an ultrahigh vacuum (10^–10^ bar).

### Electrochemistry

Cyclic voltammetry (CV), differential pulse voltammetry (DPV), and rotating disk electrode (RDE) voltammetry were performed in CH_2_Cl_2_ solution with 1 mM analyte concentration and 0.25 M Bu_4_NPF_6_ support electrolyte. For all measurements, a Pt/Ti electrode was used as the counter electrode, and leakless Ag/AgCl was used as the reference electrode. A 1.0 mm diameter glassy carbon was used for CV/DPV studies and 3.0 mm diameter glassy carbon electrodes for RDE. Measurements were performed at room temperature in spectroscopic grade CH_2_Cl_2_ and the ferrocene/ferrocenium couple was used as the internal reference.

### Density Function Theory Calculations

All calculations were performed with Orca^82−84^ suite (version 5.0.2) quantum chemistry program on STFC SCARF HPC. The scalar relativistic effect was treated with the Second Order Douglas–Kroll–Hess (DKH) method.^85^ The hybrid B3LYP functional, including D3 dispersion corrections, was employed for the electronic structure calculations. The basis set SARC2-DKH-QZVP with quadruple-ζ quality was used explicitly for Ce, and the def2-TZVP basis set of the Karlsruhe group with triple-ζ quality was used for N and O atoms, while def2-SVP basis set with double-ζ quality was used for all other elements.^86,87^ A solvation model of CPCM(Chloroform)^88^ was included for the best simulation of experimental conditions. TDDFT calculations were conducted to simulate UV–vis absorption spectra. The ORCA_MAPSPC module was used to apply a spectral broadening of 2500 cm^–1^ to the calculated excitations for the best match with experimental data. The molecular orbitals generated from orca output files were visualized using the Avogadro molecular viewing software with a default iso-surface value of 0.02. Root analysis was conducted and compared for all TDDFT simulations until the saturation of all measurable experimental features. Natural population analysis was conducted using NBO 7.0 in Orca.^89^ DFT calculations were performed both using crystallographic coordinates and following geometry optimization. The TDDFT UV–vis simulations best reproduced the experimental spectra when crystallographic coordinates. Within this text, the presented DFT analysis is based on crystallographic data. DFT analysis based on geometry-optimized structures is available in the Supporting Information.

## Supporting Information Available

Additional TGA, powder X-ray diffraction, crystallographic and structural data, structural analysis, IR, magnetic measurements, UV–vis-NIR, and DFT (PDF)
DFT-optimized XYZ coordinates of complexes (ZIP)
