# Supplementary material for: Tuning the Electronic Properties of Tetravalent Cerium Complexes via Ligand Derivatization
Source: Inorg Chem. 2025 Mar 24;64(13):6519–30. doi: 10.1021/acs.inorgchem.4c05371 (PMC11979883; doi:10.1021/acs.inorgchem.4c05371)
Supplement: Supplementary file 1 — ic4c05371_si_001.pdf [file ic4c05371_si_001.pdf]

Supporting Information to accompany

Tuning the electronic properties of tetravalent cerium complexes via ligand derivatization

Georgilett Pérez Bedwell [a], Nithin Suryadevara [a], Zhibo Qi [b,c], Robert W. Gable [a], Peter Bencok [d], Michael L. Baker [b,c*] and Colette Boskovic [a*]

[a] *School of Chemistry, University of Melbourne, Parkville, Victoria 3010, Australia.*

[b] *Department of Chemistry, The University of Manchester, Manchester, M13 9PL, UK.*

[c] *The University of Manchester at Harwell, Diamond Light Source, Harwell Campus, OX11 0DE, UK.*

[d] *Diamond Light Source, Harwell Science and Innovation Campus, Chilton, Didcot, OX11 0DE, UK.*

[*] *Email: c.boskovic@unimelb.edu.au ; michael.baker@manchester.ac.uk*

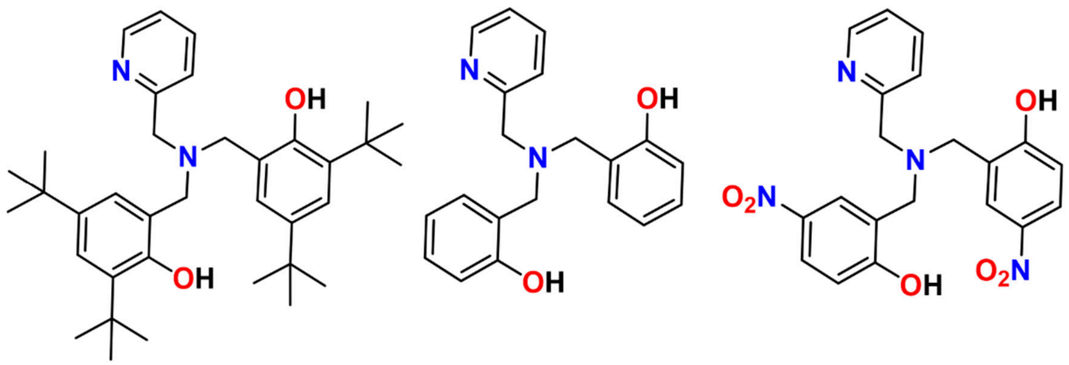

Figure S1. Proligands H_2L_{tBu} (left) H_2L_H (centre) H_2L_{NO2} (right).

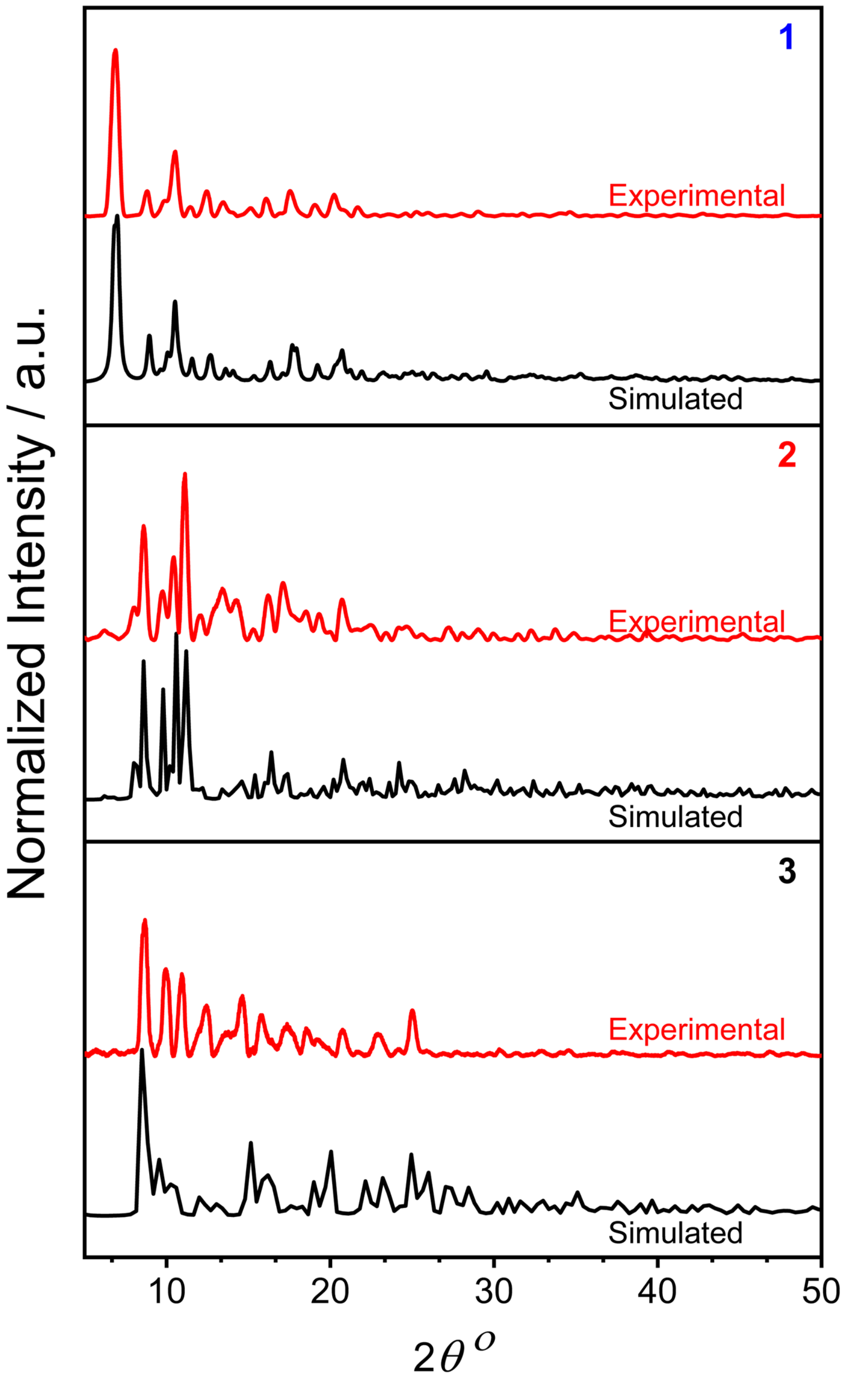

Figure S2. Powder X-ray diffraction patterns for **1** (top), **2** (middle) and **3**·$0.8CH_2Cl_2 \cdot 1.5H_2O$ (bottom) collected at 100 K (red) and simulated from the crystal structures at 100 K (black).

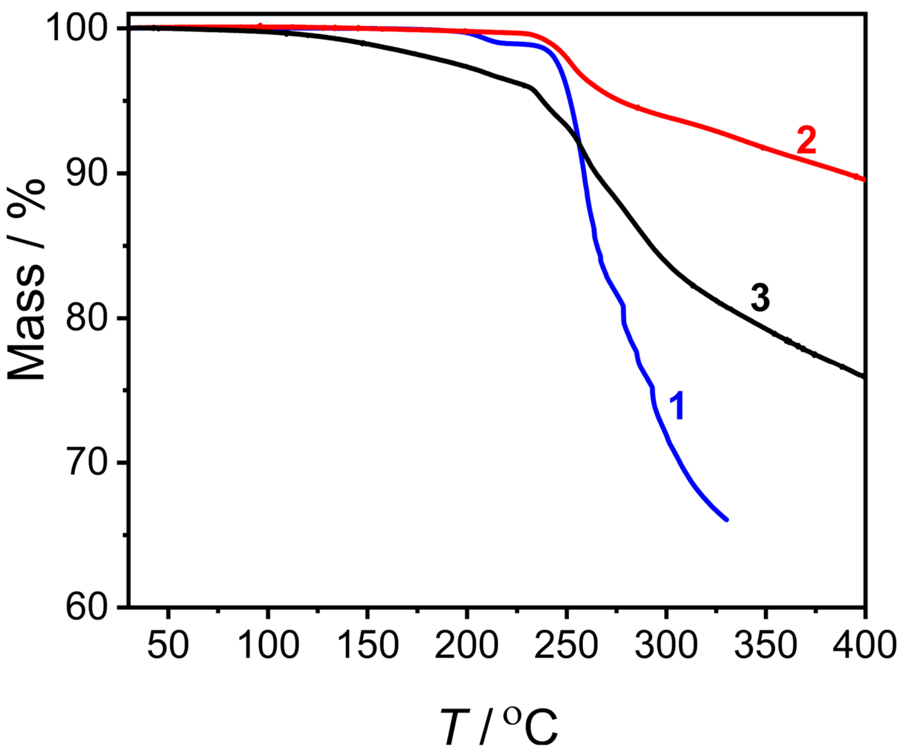

Figure S3. Thermogravimetric analysis date for **1** (blue), **2** (red) and **3**$\cdot 0.8CH_2Cl_2 \cdot 1.5H_2O$ (black).

Table S1. Crystal data for **1**, **2** and **3·0.75CH$_2$Cl$_2$·H$_2$O**.

	**1**	**2**	**3·0.75 CH$_2$Cl$_2$·H$_2$O**
Empirical formula	$C_{72}H_{100}CeN_4O_4$	$N_4O_4CeC_{40}H_{36}$	$C_{40}H_{34}CeN_8O_{12}$ ·0.75CH_2Cl_2·H_2O
Formula weight	1225.67	776.85	1038.56
Temperature/K	100.00(10)	99.98(12)	100.00(10)
Crystal system	Monoclinic	Monoclinic	Monoclinic
Space group	*I*2/*a*	*P*2$_1$/*c*	*P*2$_1$/*c*
a/Å	19.4284(8)	17.26855(12)	19.7579(2)
b/Å	17.2864(6)	20.38901(16)	10.42852(11)
c/Å	20.8987(9)	19.43521(12)	20.8429(2)
α/°	90	90	90
β/°	109.190(5)	91.8244(6)	92.4986(11)
γ/°	90	90	90
Volume/Å^3	6628.7(5)	6839.45(8)	4290.51(8)
Z	4	8	4
Z'	0.5	2	1
ρ_{calc}g/cm^3	1.228	1.509	1.608
μ/mm^{-1}	5.681	10.671	9.704
F(000)	2600	3152	2094
Radiation	Cu Kα (λ = 1.54184)	Cu Kα (λ = 1.54184)	Cu Kα (λ = 1.54184)
2Θ range for data collection/°	7.026 to 153.400	5.120 to 152.726	8.492 to 160.700
Index ranges	$-23 \le h \le 24$, $-20 \le k \le 21$, $-26 \le l \le 23$	$-21 \le h \le 21$, $-25 \le k \le 25$, $-24 \le l \le 20$	$-25 \le h \le 24$, $-13 \le k \le 11$, $-26 \le l \le 25$
Reflections collected	30450	75232	61208
Independent reflections	6746 [R_{int} = 0.1292, R_{sigma} = 0.0928]	13955 [R_{int} = 0.0704, R_{sigma} = 0.0467]	9303 [R_{int} = 0.0883, R_{sigma} = 0.0530]
Data/restraints/ parameters	6746/0/378	13955/0/883	9303/12/550
Goodness-of-fit on F^2	1.053	1.035	1.033
Final R indexes [$I >= 2\sigma(I)$]	R1 =0.0631, wR2 =0.1303	R1 = 0.0303, wR2 = 0.0692	R_1 = 0.0418, wR_2 = 0.0989
Final R indexes [all data]	R1 =0.0828, wR2 =0.1388	R1 = 0.0361, wR2 = 0.0723	R_1 = 0.0497, wR_2 = 0.1031
Largest diff. peak/hole / e Å^{-3}	1.28 /-1.52	0.67 /-0.57	0.93 /-0.65
CCDC No.	2408522	2408521	2408520

Table S2. Shape indices for 8-coordinate Ce atoms in **1**, **2** and **3**·0.75CH_2Cl_2·H_2O.

	**1**	**2**[a]	**2**[b]	**3·0.75CH_2Cl_2·H_2O**
**OP-8**	28.205	29.169	29.529	30.042
**HPY-8**	23.154	23.660	25.208	25.176
**HBPY-8**	15.332	15.230	18.138	18.145
**CU-8**	12.013	12.143	12.699	12.286
**SAPR-8**	3.269	3.312	3.877	3.846
**TDD-8**	2.395	2.404	1.450	1.233
**JGBF-8**	11.163	10.513	10.847	11.058
**JETBPY-8**	24.284	25.012	25.616	26.434
**JBTPR-8**	2.336	1.399	2.043	2.294
**BTPR-8**	3.067	2.118	2.867	3.005
**JSD-8**	1.122	1.082	0.495	0.546
**TT-8**	12.843	12.839	12.108	11.829
**ETBPY-8**	22.260	23.813	24.366	24.773

SHAPE index geometry for an octacoordinated metal complex in SHAPE 2.1. A value of 0 represents a perfect geometry with no distortion. OP-8: Octagon; HPY-8: Heptagonal pyramid; HBPY-8: Hexagonal bipyramid; CU-8: Cube; SAPR-8: Square antiprism; TDD-8: Triangular dodecahedron; JGBF-8: Johnson gyrobifastigium J26; JETBPY-8: Johnson elongated triangular bipyramid J14; JBTPR-8: Biaugmented trigonal prism J50; BTPR-8: Biaugmented trigonal prism; JSD-8: Snub diphenoid J84; TT-8: Triakis tetrahedron; ETBPY-8: Elongated trigonal bipyramid.

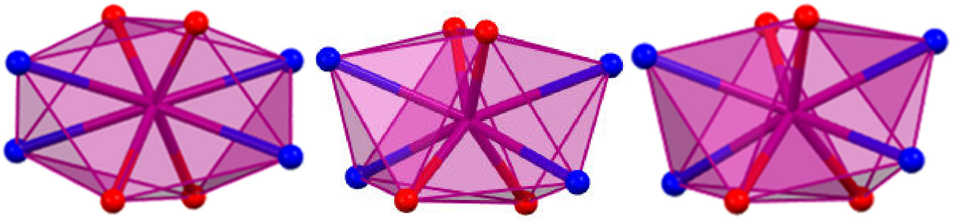

Figure S4. Polyhedral representation for **1** (left), **2** (centre) and **3** (right) illustrating the snub disphenoid (JSD-8:) geometry as determined via SHAPE 2.1.

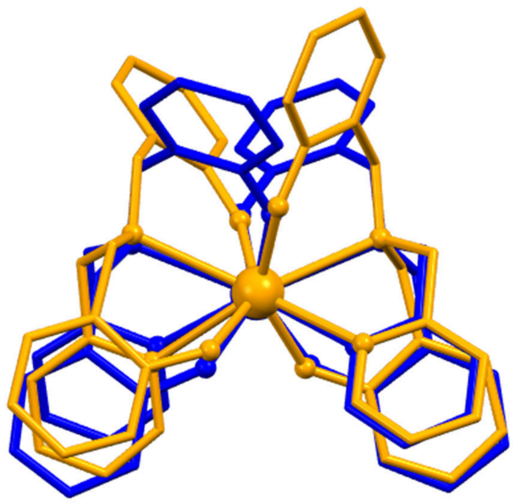

Figure S5. Molecular structure of **2** as determined via single-crystal X-ray diffraction at 100 K. Two individual molecules **2**[a] (yellow), and **2**[b] (blue) present in the asymmetric unit are overlayed to display the structural differences. Hydrogen atoms have been omitted for clarity.

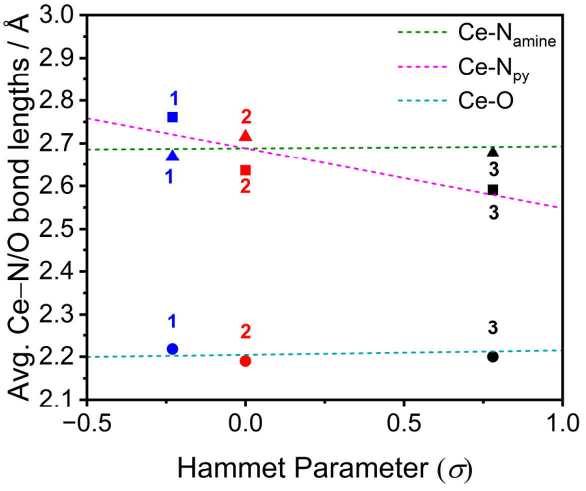

Figure S6. Correlation between average Ce–N$_{amine}$ (▲), Ce–N$_{py}$ (■) and Ce–O (●) bond lengths from crystallographic data versus the Hammett *σ* parameters for the ligand aryl substituents: 2,4–^{t}Bu (**1**), H (**2**), 4–NO_2 (**3**).[3] Error bars are contained within the symbols. Dashed lines represent lines of best fit (R^2 = 0.01 for Ce–N$_{amine}$, 0.71 for Ce–N$_{py}$, 0.14 for Ce–O).

Table S3. Bond valence sum (BVS) values for **1**, **2** and **3**·0.75CH_2Cl_2·H_2O.

		**Ce^{III}**		
	**1**	**2**[a]	**2**[b]	**3**·0.75CH_2Cl_2·H_2O
**Ce-O1**	0.82	0.83	0.85	0.78
**Ce-O2**	0.71	0.80	0.78	0.85
**Ce-O3**	0.82	0.80	0.85	0.78
**Ce-O4**	0.71	0.83	0.84	0.80
**Ce-N1**	0.25	0.28	0.28	0.31
**Ce-N2**	0.32	0.39	0.31	0.40
**Ce-N3**	0.25	0.28	0.30	0.32
**Ce-N4**	0.32	0.37	0.34	0.40
**BVS Value**	**4.21**	**4.60**	**4.54**	**4.64**
		**Ce^{IV}**		
**Ce-O1**	0.62	0.71	0.75	0.69
**Ce-O2**	0.72	0.73	0.68	0.74
**Ce-O3**	0.62	0.71	0.75	0.69
**Ce-O4**	0.72	0.73	0.73	0.70
**Ce-N1**	0.28	0.25	0.24	0.27
**Ce-N2**	0.22	0.34	0.27	0.35
**Ce-N3**	0.28	0.25	0.26	0.23
**Ce-N4**	0.22	0.33	0.30	0.35
**BVS Value**	**3.70**	**4.04**	**3.99**	**4.07**

BVS: Bond valence sum calculations assuming a trivalent and tetravalent oxidation states for cerium metal centre using the bond lengths reported on Table 1 (R_{ij}). BSV = $\sum_j s_{ij}$, where s_{ij} =exp[(R_o-R_{ij})/b].[1] For cerium complexes with coordination number equal to eight, constant R_0 was set as 2.118 Å (Ce^{III}) and 2.070 Å (Ce^{IV}) for Ce-O bonds.[1,2] Likewise in the case of Ce-N bonds, the assigned values correspond to 2.251 Å (Ce^{III}) and 2.202 Å (Ce^{IV}). As for constant b, the accepted value of 0.370 was used.[2]

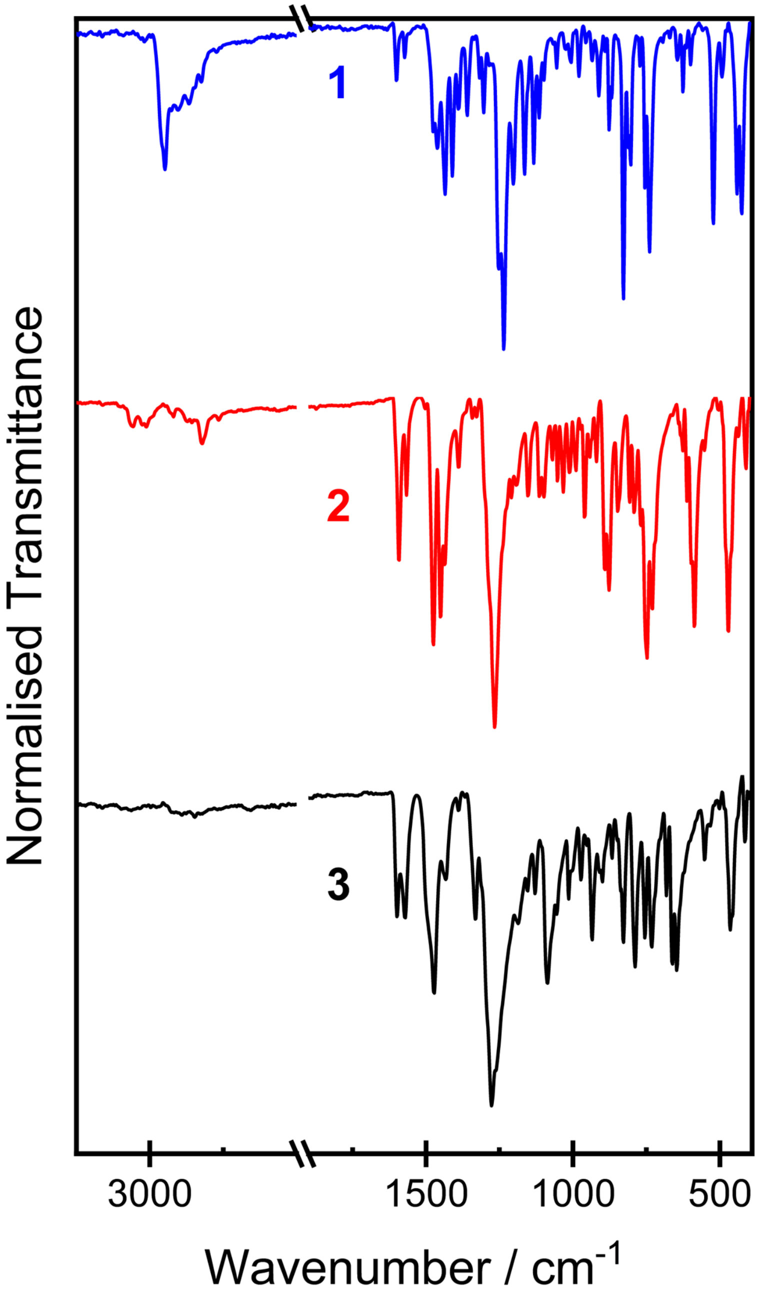

Figure S7. Infrared (ATR) spectra of **1** (blue), **2** (red) and **3**·0.8 CH_2Cl_2·1.5H_2O (black).

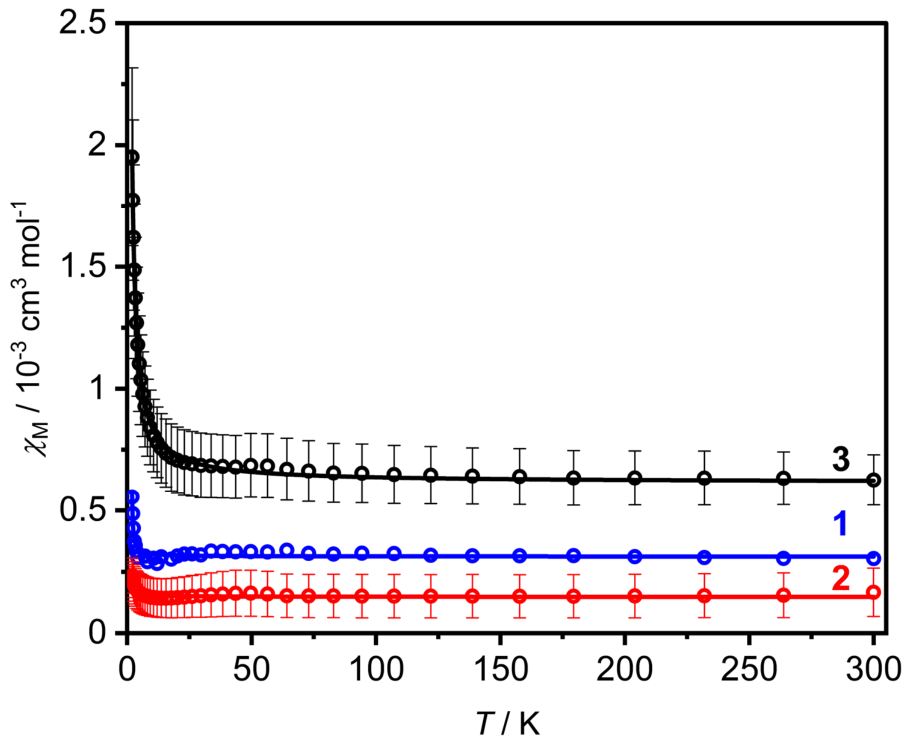

Figure S8. Plots of χ_M versus T for **1** (blue), **2** (red), **3**·0.8 CH_2Cl_2·1.5H_2O (black) with an applied field of 0.1 T; error bars arise from averaging 2-3 data sets. The line is the fit to the Curie–Weiss law + TIP as described in the text.

Table S4. Fit parameters for χ_M vs T plots in the range 2-300 K for **1**, **2** and **3**·0.8 CH_2Cl_2·1.5H_2O

**Compound**	**1**	**2**	**3·0.8CH₂Cl₂·1.5H₂O**
C_J (emu·K·mol^{-1})	(8.77 ± 2.16) x 10^{-5}	(7.09 ± 1.19) x 10^{-5}	(2.18 ± 0.005) x 10^{-3}
θ_{CW} (K)	1.66 ± 0.093	1.22 ± 0.151	0.396 ± 0.0400
χ_{TIP} (emu·mol^{-1})	(3.12 ±0.06) x 10^{-4}	(1.48 ± 0.02) x 10^{-4}	(6.15 ± 0.04) x 10^{-4}
Ce^{III} impurity (%)	0.011	0.0087	0.270
R^2	0.87	0.93	0.99

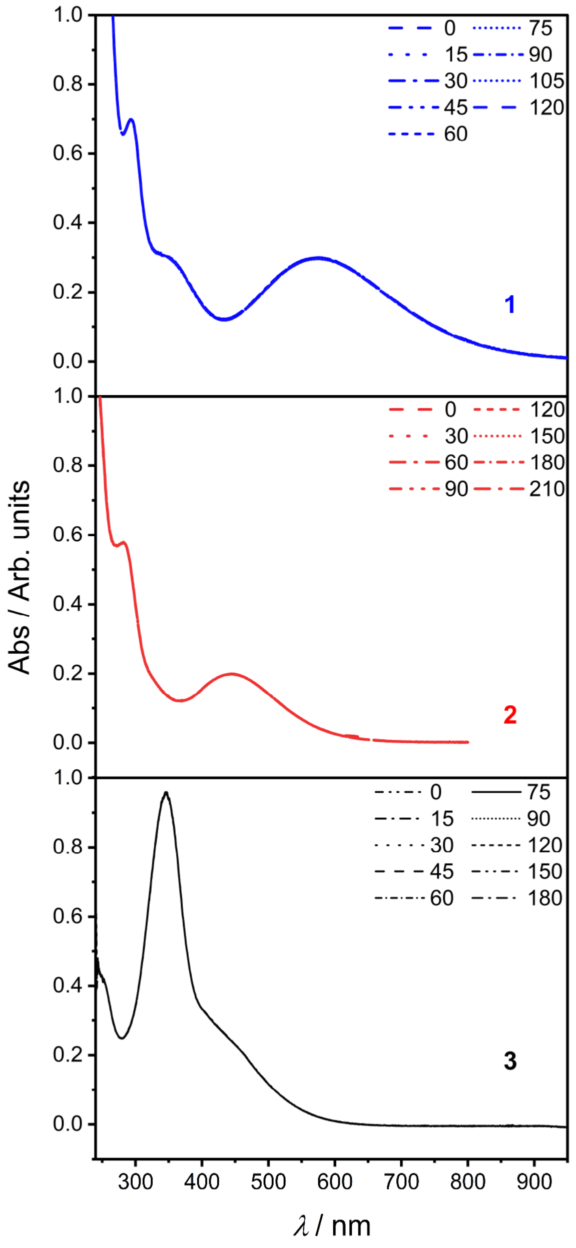

Figure S9. Electronic absorption spectra for **1** (top), **2** (top centre), and **3** (down) in $CHCl_3$ over time (in minutes).

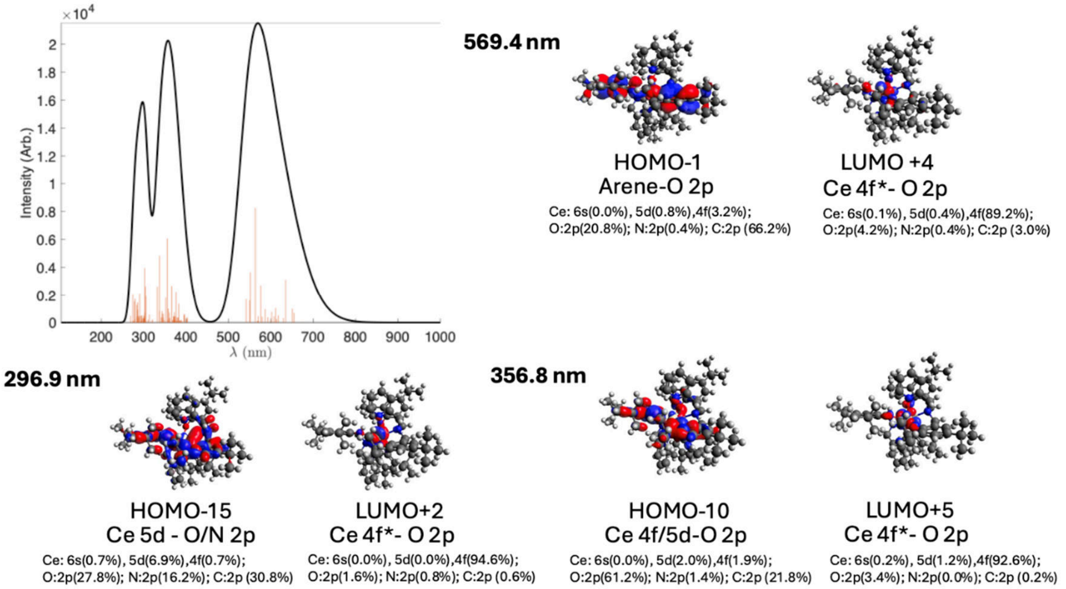

Figure S10. The UV-vis TDDFT simulation for XRD structure of **1** and molecular orbitals corresponding with TDDFT transitions that carry large transition oscillator strengths.

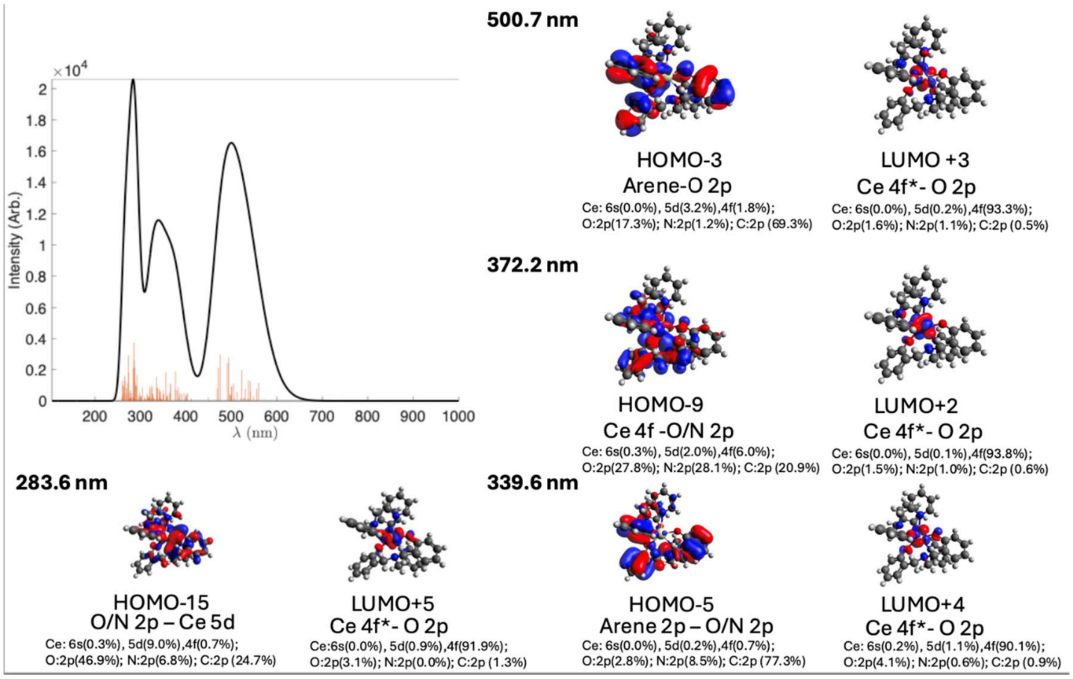

Figure S11. The UV-vis TDDFT simulation for XRD structure of **2** and molecular orbitals corresponding with TDDFT transitions that carry large transition oscillator strengths.

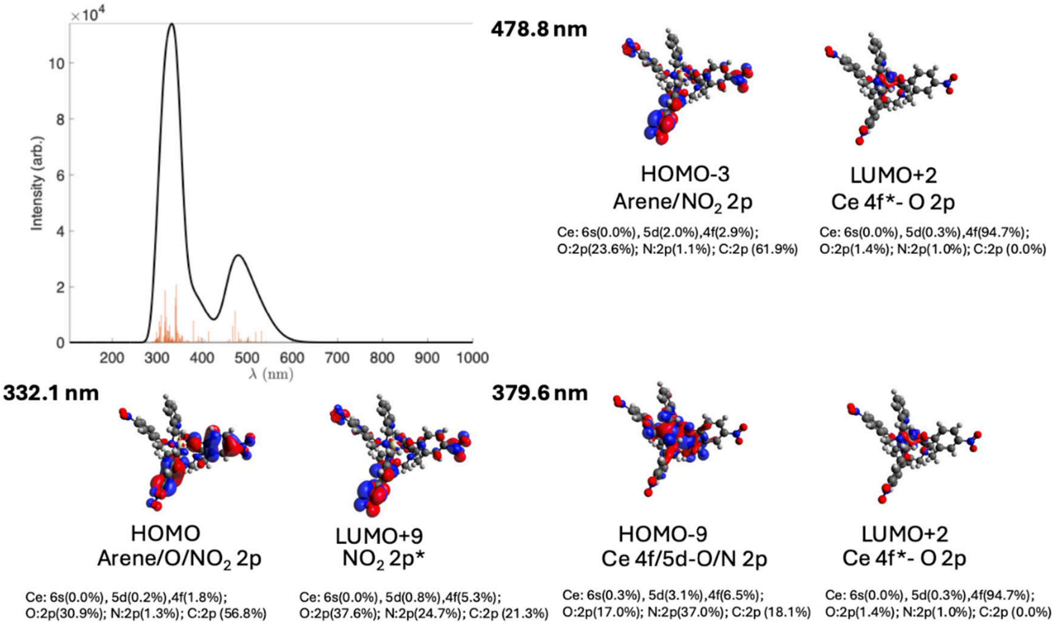

Figure S12. The UV-vis TDDFT simulation for XRD structure of **3** and molecular orbitals corresponding with TDDFT transitions that carry large transition oscillator strengths.

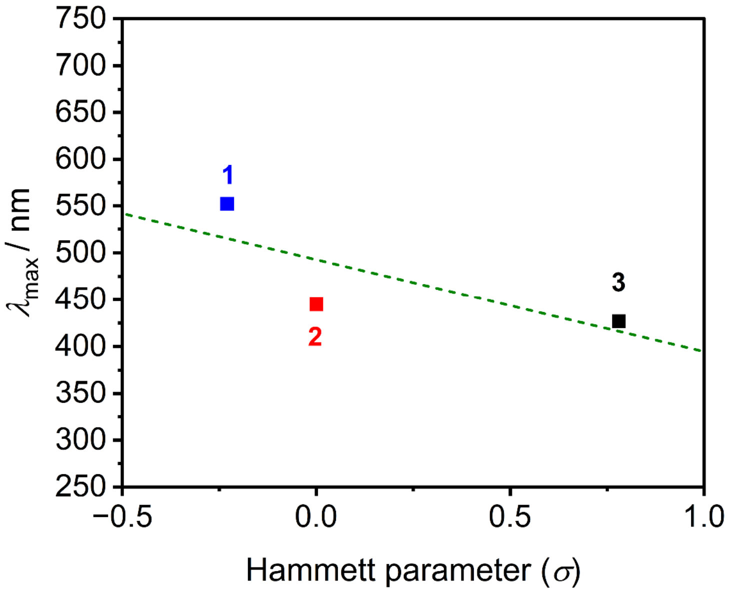

Figure S13. Correlation between the experimental λ_{max} versus the Hammett σ parameters for the aryl substituents: 2,4–^{t}Bu (**1**), H (**2**), 4–NO_2 (**3**).[3] Dashed line represent the linear fit (R^2 = 0.590).

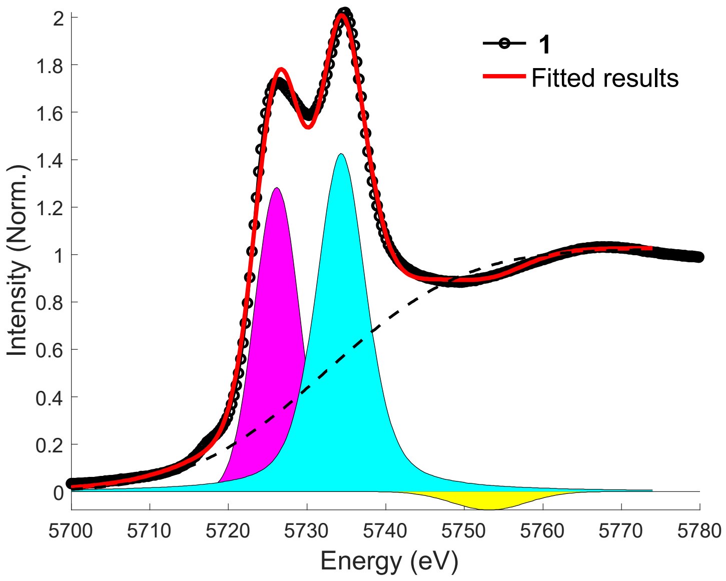

Figure S14. Ce L_3-edge XANES peak fitting analysis for **1**.

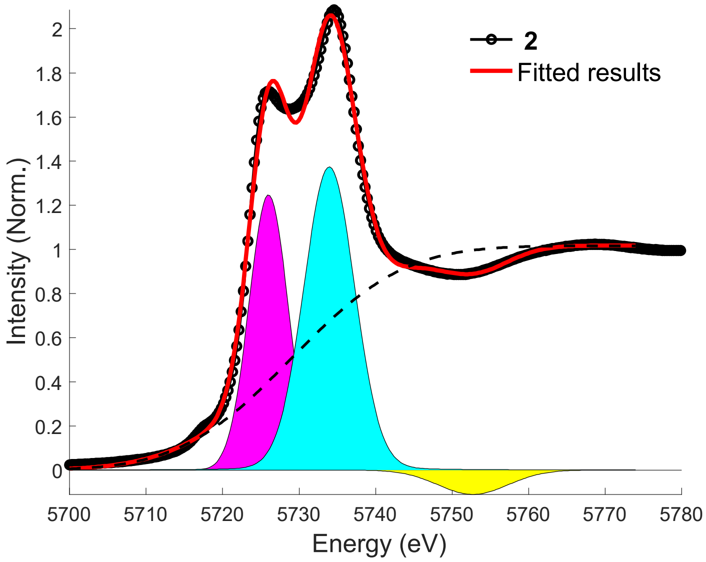

Figure S15. Ce L_3-edge XANES peak fitting analysis for **2**.

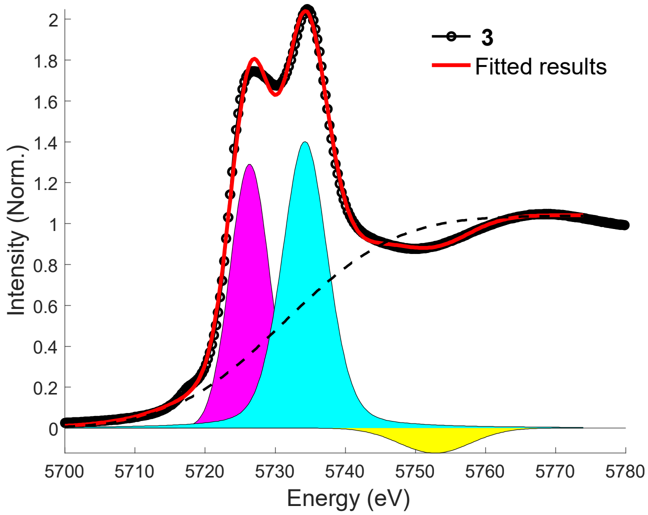

Figure S16. Ce L_3-edge XANES peak fitting analysis for **3**.

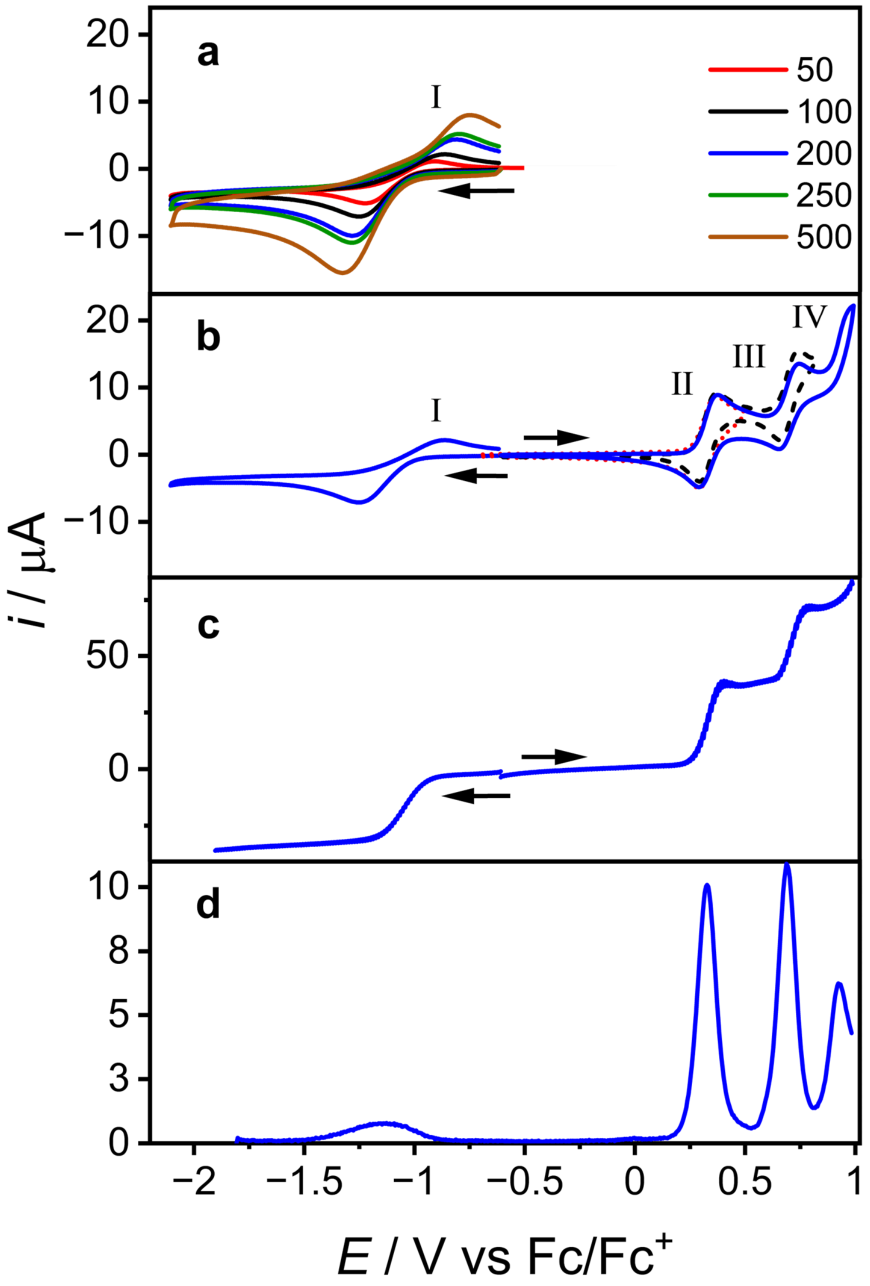

Figure S17. Cyclic (a and b), rotating disk electrode (c) and differential pulse (d) voltammograms for **1** in 1.0 mM DCM solution with 0.25 M Bu_4NPF_6 as supporting electrolyte. (a) at indicated scan rates (mV s^{-1}); (b) at scan rate of 100 mV s^{-1} (c) RDE at 500 rotations per minute and (d) DPV at a scan rate of 10 mV s^{-1} with a pulse width of 500 ms.

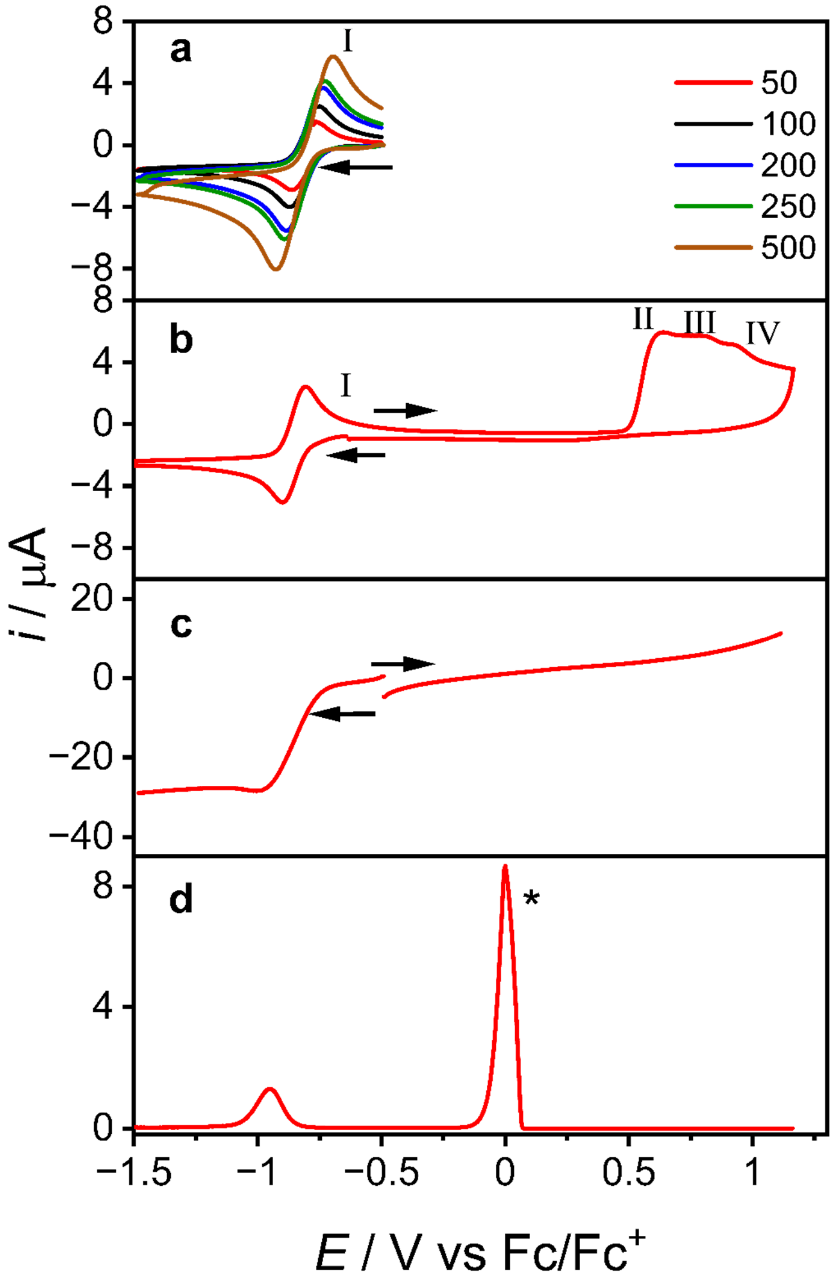

Figure S18. Cyclic (a and b), rotating disk electrode (c) and differential pulse (d) voltammograms for **2** in 1.0 mM DCM solution with 0.25 M Bu_4NPF_6 as supporting electrolyte. (a) at indicated scan rates (mV s^{-1}); (b) at scan rate of 100 mV s^{-1} (c) RDE at 500 rotations per minute and (d) DPV at a scan rate of 10 mV s^{-1} with a pulse width of 500 ms. Fc/Fc^+ Redox potential (*).

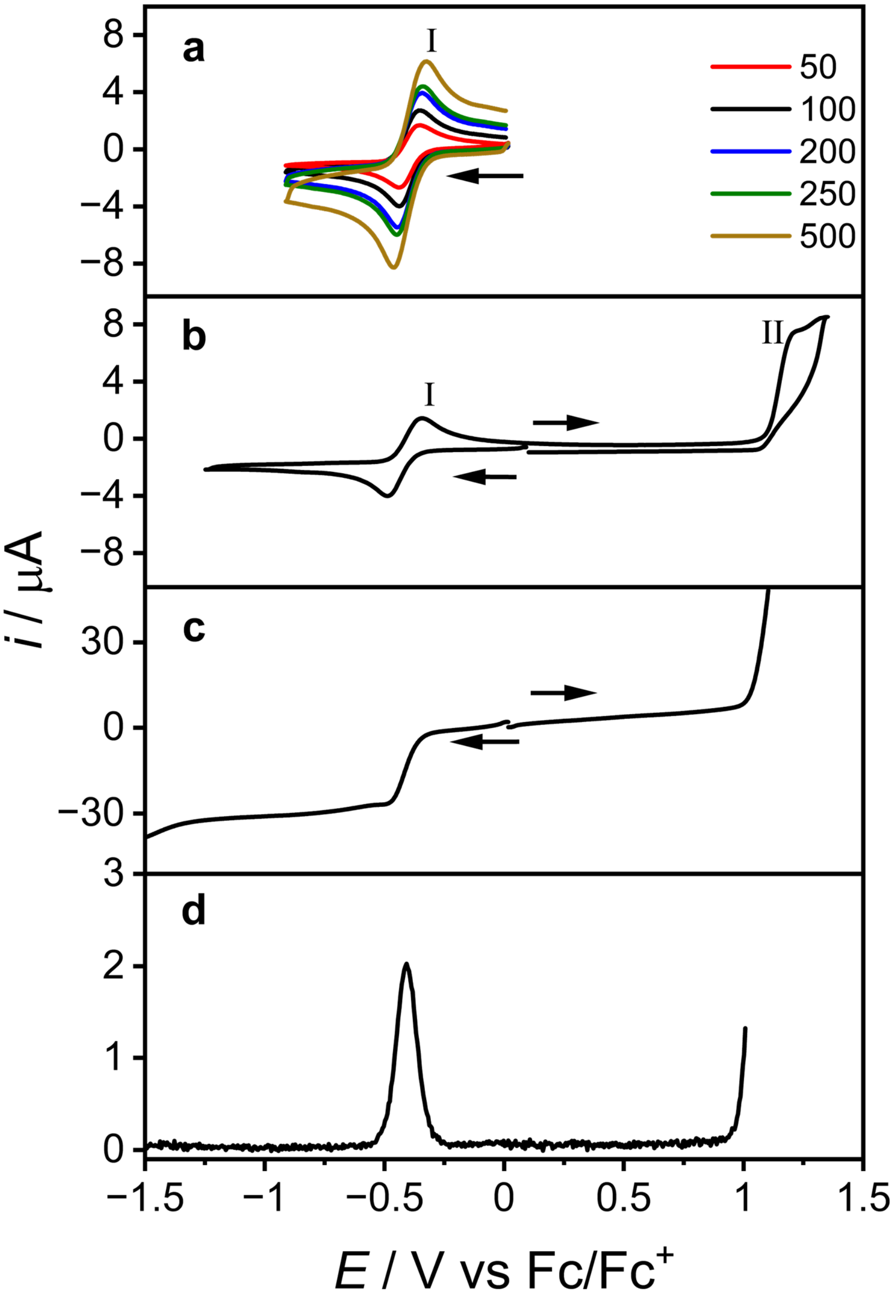

Figure S19. Cyclic (a and b), rotating disk electrode (c) and differential pulse (d) voltammograms for **3** in 1.0 mM DCM solution with 0.25 M Bu_4NPF_6 as supporting electrolyte. (a) at indicated scan rates (mV s^{-1}); (b) at scan rate of 100 mV s^{-1} (c) RDE at 500 rotations per minute and (d) DPV at a scan rate of 10 mV s^{-1} with a pulse width of 500 ms.

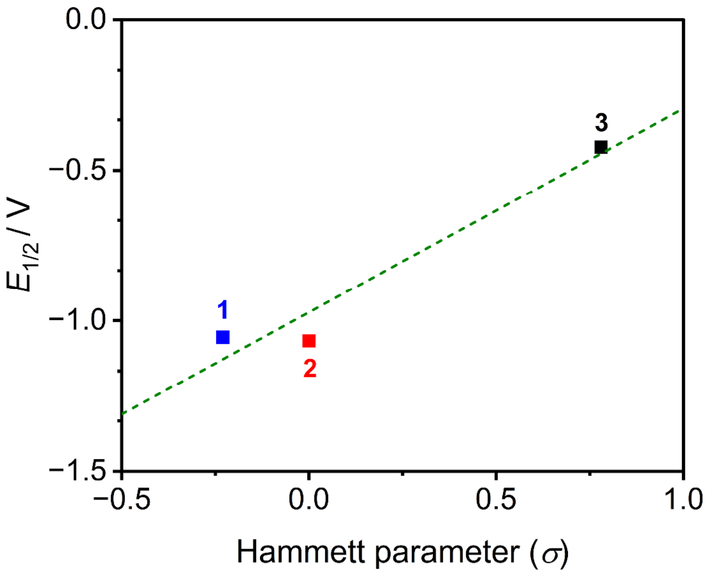

Figure S20. Correlation between the experimental $E_{1/2}$ versus the Hammett σ parameters for the aryl substituents: 2,4–^{t}Bu (**1**), H (**2**), 4–NO_2 (**3**).[3] Dashed line represents the linear fit (R^2 = 0.945).

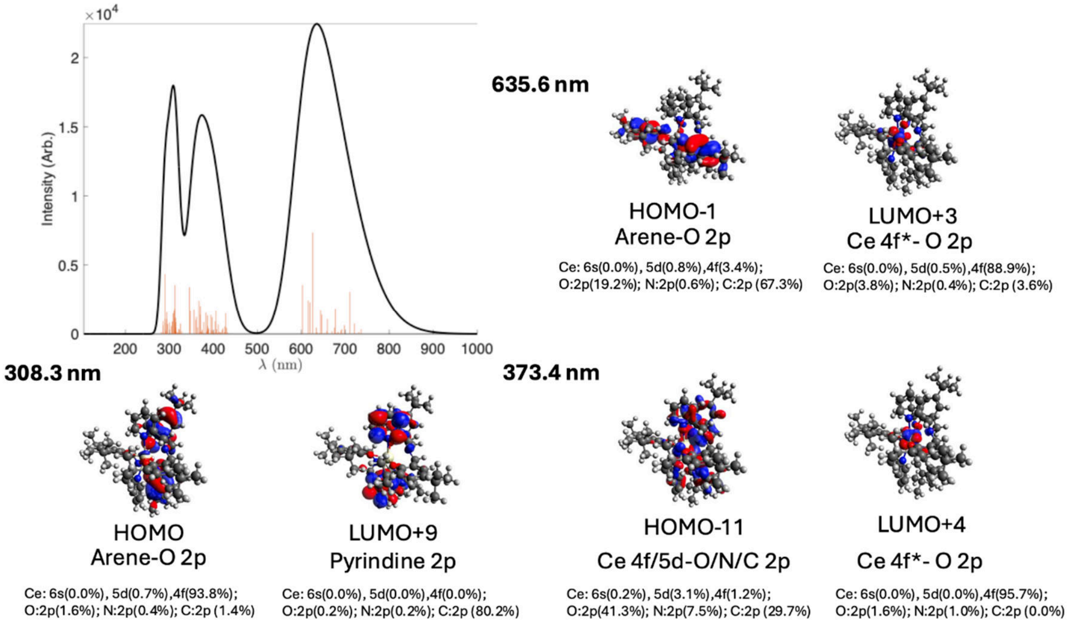

Figure S21. The UV-vis TDDFT simulation for geometry optimized structure of **1** and molecular orbitals corresponding with TDDFT transitions that carry large transition oscillator strengths.

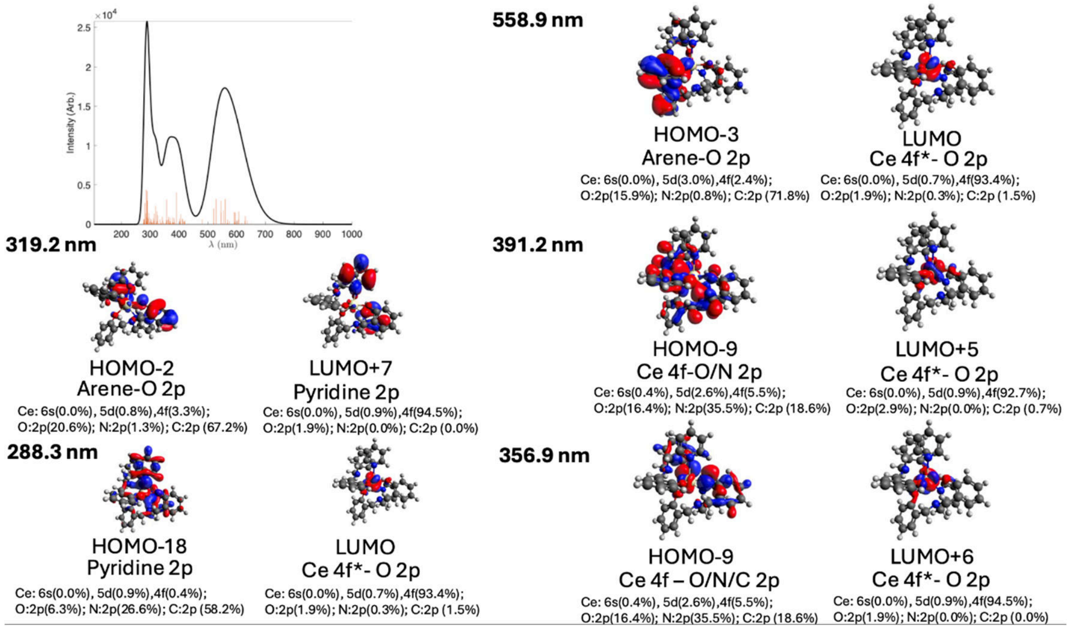

Figure S22. The UV-vis TDDFT simulation for geometry optimized structure of **2** & molecular orbitals corresponding with TDDFT transitions that carry large transition oscillator strengths.

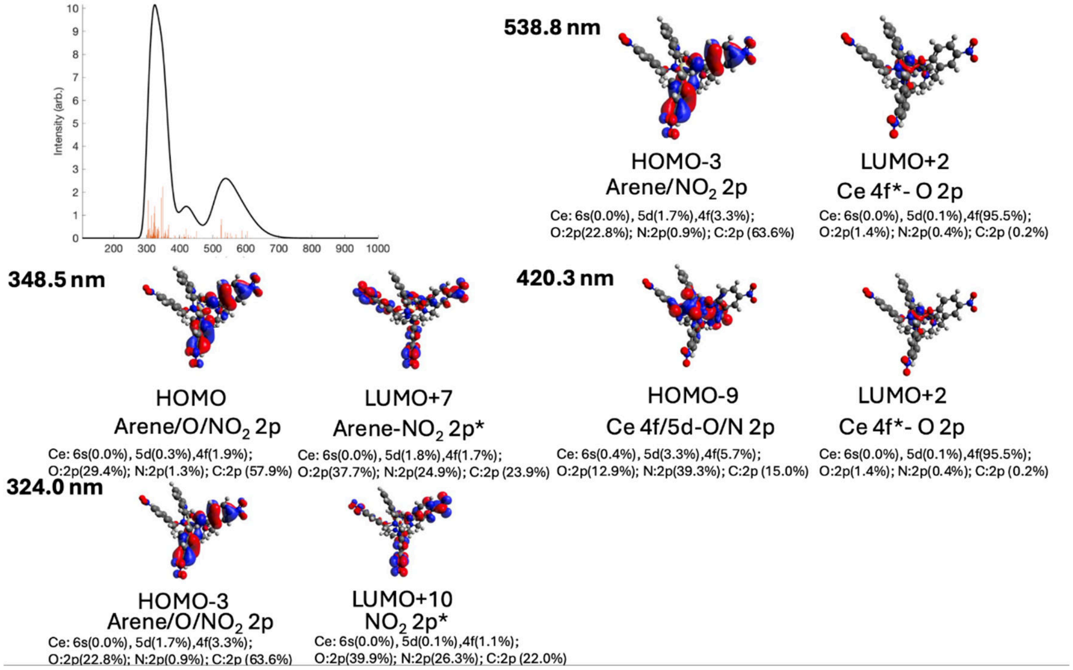

Figure S23. The UV-vis TDDFT simulation for geometry optimized structure of **3** and molecular orbitals corresponding with TDDFT transitions that carry large transition oscillator strengths.

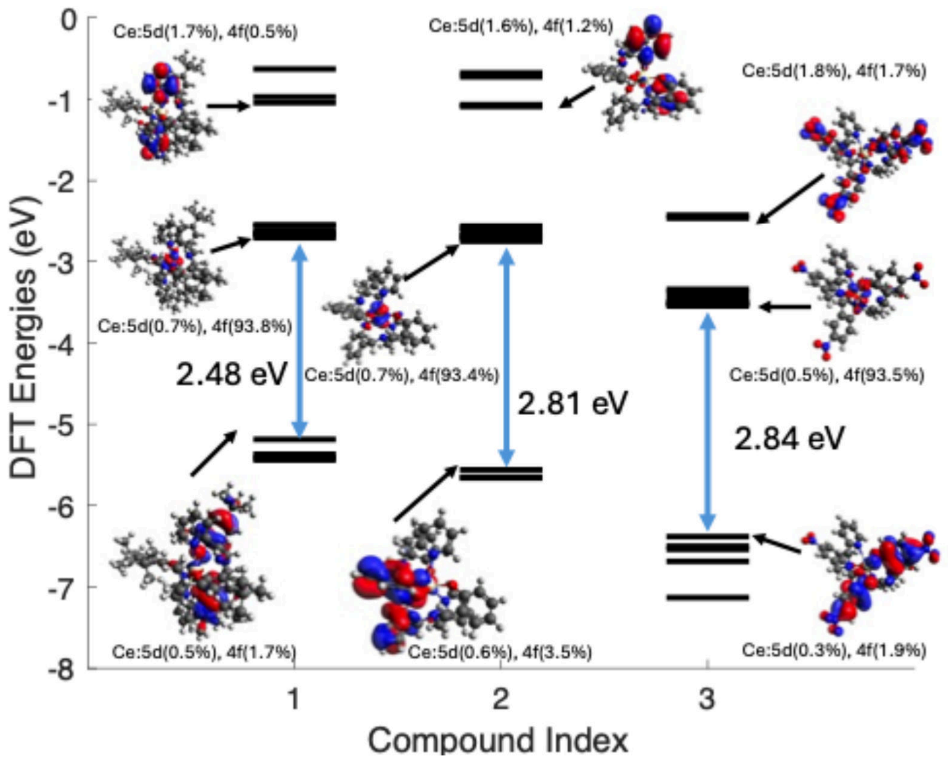

Figure S24. Valence molecular orbital diagrams for **1**, **2**, **3** obtained from DFT calculations based on geometry optimized structure. The calculated HOMO-LUMO energy gap is 2.48 eV for **1**, 2.81 eV for **2** and 2.84 eV for **3**.

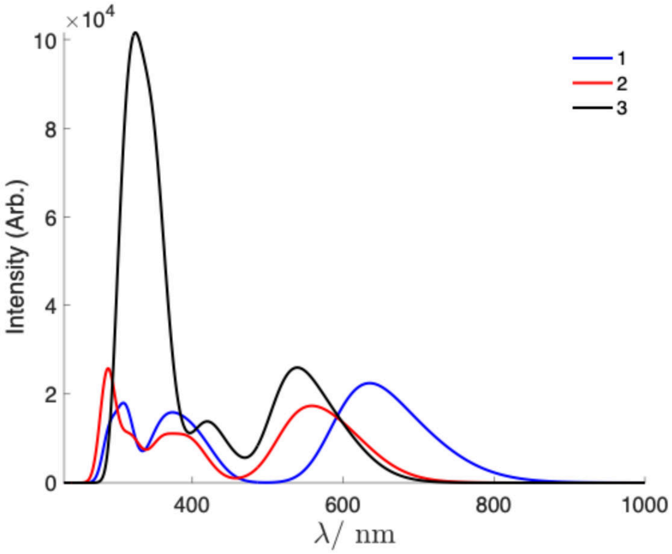

Figure S25. TDDFT calculated UV-vis spectra based on geometry optimized structures.

Table S5. Electronic spectral data (λ /nm (*ε* / L·mol^{-1}·cm^{-1}) in $CHCl_3$ and the solid state for **1**, **2** and **3**. TDDFT transitions and oscillator strengths were computed based on geometry optimized structures.

**1**			**2**			**3**			**Assignment**
Solution	Solid	Calc.	Solution	Solid	Calc.	Solution	Solid	Calc.	
552 (3.45 × 10^3)	558	635.6 (0.0847)	445 (7.33 × 10^3)	464	558.9 (0.0368)	~437 (9.86 × 10^3)	442	538.8 (0.0958)	**1**: aryloxide O lone pair → O 2*p* - Ce 4*f** (LMCT)<br><br>**2:** aryloxide O lone pair → O 2*p* - Ce 4*f** (LMCT)<br><br>**3**: NO_2-aryloxide O lone pair → O 2*p* - Ce 4*f** (LMCT)
						407 (1.18 ×10^4)	416(sh)	420.3 (0.0469)	O/N 2*p* – Ce 4f/5d → O 2*p* - Ce 4*f** (LMCT)
343 (3.80 × 10^3)	356	373.4 (0.0279)	335 (6.09 × 10^3)	358, 370	356.9 (0.0363), 391.2 (0.0466)	347 (3.81 × 10^4)	366	324.0 (0.190), 348.5 (0.258)	**1:** O/N/C 2*p* – Ce 4f/5d → O 2*p* - Ce 4*f** (LMCT)<br><br>**2**: O/N 2*p* - Ce 4*f* → O 2*p* - Ce 4*f** (LMCT)<br><br>**3**: arene – NO_2/O π → NO_2/arene π* (LLCT)
286 (1.06 × 10^4)	260, 292	308.3 (0.0409)	282 (2.33 × 10^4)	290	319.2 (0.0269)<br><br>288.3 (0.0482)				**1**: arene – O π → Pyrindine π* (LLCT)<br><br>**2**: arene – O π → Pyrindine π* (LLCT) & Pyrindine π → O 2*p* - Ce 4*f** (LMCT)

Table S6. The Ce effective *4f* (n_{4f}), *5d* (n_{5d}) and *6s* (n_{6s}) electron occupation obtained from DFT natural atomic orbital occupancy, based on geometry-optimized structures.

	**1**	**2**	**3**·0.8 CH_2Cl_2·1.5H_2O
DFT n_{6s}	0.09	0.12	0.12
DFT n_{5d}	0.53	0.89	0.85
DFT n_{4f}	0.83	0.83	0.82

References

(1) Roulhac, P. L.; Palenik, G. J. Bond Valence Sums in Coordination Chemistry. The Calculation of the Oxidation State of Cerium in Complexes Containing Cerium Bonded Only to Oxygen. *Inorg. Chem.* **2003**, *42*, 118–121.

(2) Palenik, G. J.; Hu, S. -Z. Assignment of Oxidation States in Metal Complexes. Cerium(III) or Cerium(IV) and Other Questions. *Inorg. Chim. Acta* **2009**, *362*, 4740–4743.

(3) Hansch, Corwin.; Leo, A.; Taft, R. W. A Survey of Hammett Substituent Constants and Resonance and Field Parameters. *Chem. Rev.* **1991**, *91*, 165–195.
